# Examining the low uptake of LARC in Ethiopia: an analysis of individual-level predisposing and enabling factors

**DOI:** 10.3389/fgwh.2025.1547891

**Published:** 2025-06-06

**Authors:** Mintesnot T. Teni, Travis Loux, Ness Sandoval, Anne Sebert Kuhlmann

**Affiliations:** ^1^ College for Public Health and Social Justice, Saint Louis University, St. Louis, MO, United States; ^2^ College of Arts and Sciences, Saint Louis University, St. Louis, MO, United States

**Keywords:** long acting reversible contraception (LARC), Ethiopia, predisposing factors, enabling factors, Andersen's Behavioral Model, contraceptive use

## Abstract

**Introduction:**

According to the 2019 Ethiopian Demographic and Health Survey (EDHS) only 11% of married reproductive-age women in Ethiopia use long-acting reversible contraceptives (LARCs). This study aimed to identify individual characteristics associated with LARC uptake compared to short-acting contraceptives, traditional and barrier methods, and non-contraceptive use.

**Methods:**

Data from the 2019 Performance Monitoring for Action (PMA) Ethiopia survey (*n* = 8,182) were used to run multilevel logistic regression models. The sample includes sexually active reproductive-age women (15–49 years). Independent variables were grouped into predisposing and enabling factors guided by the Andersen Behavioral Model of Health Services.

**Results:**

LARC uptake in this study was 9.7%. Older, single, nulliparous, and Muslim women had lower LARC use than non-contraceptive and traditional/barrier method use. When compared to short-acting method use, low LARC use was associated with smaller household size and no exposure to family planning information. When compared to all other groups, contraceptive autonomy was associated with higher LARC uptake. Younger women and women living in rural areas were less likely to use IUDs than implants.

**Discussion:**

Policymakers could use these findings to tailor interventions to specific populations with low LARC uptake. Training providers on counseling and LARC eligibility could help improve LARC uptake among populations, including less-empowered women. Involving religious leaders in contraceptive health education has the potential to increase LARC use.

## Introduction

Unmet need for family planning is one of the biggest reproductive health problems in developing countries leading to higher rates of unintended pregnancy. An estimated 214 million women have an unmet need for family planning, mainly in South Asia and Sub-Saharan countries (1). According to the Ethiopia Demographic and Health Survey (EDHS) 2016, one in four pregnancies in Ethiopia are unintended pregnancies (2). This rate is lower than the average of unintended pregnancies worldwide, some literature attributes this relatively low rate of unintended pregnancies in Ethiopia to the increase in the utilization of family planning services. Despite the relatively low rate of unintended pregnancies the economic impact and the maternal and infant morbidity and mortality burden of unintended pregnancies in Ethiopia is severe, evidenced by the high maternal (267/100,000 live births) and neonatal mortality rate (26.2/1,000) of the country (3, 4). One strategy to resolve the unmet need for family planning is to increase access to and utilization of Long-acting reversible contraception (LARC) (5–7). Greater use of highly effective contraception that is less dependent on individual users' adherence, such as LARC, can reduce unintended pregnancy rates in at-risk populations (8).

LARC methods provide reliable, long-term, highly effective pregnancy prevention after one-time placement of a device. Increased use of LARC can profoundly impact the rates of unintended pregnancies and abortions and may improve the reproductive health outcomes of women (9–12). According to the World Health Organization (WHO), the effectiveness of LARC is significantly higher than short-term contraceptive methods; only one unintended pregnancy occurs out of 2,000 LARC users in the first year of use compared to more than 100 unintended pregnancies per 2,000 short-acting contraceptives [pills and depo medroxyprogesterone acetate (DMPA) injectable] users (13). Furthermore, a recent modeling study showed the role LARCs could play in preventing unintended pregnancies by predicting that if 20% of sub-Saharan African women using pills or injectables switch to LARCs, it could prevent 1.8 million unintended pregnancies, more than 500,000 abortions, and 10,000 maternal deaths across the region over a five-year period (14). This evidence suggests the importance of switching to a more effective and reliable contraception method, such as LARC, to improve maternal, sexual, and reproductive health outcomes.

LARC use worldwide is low compared to other methods, with only 9% and 18% of all reproductive-age women (15–49 years) using LARCs in developed and developing countries, respectively (15). Ethiopia has one of the highest LARC uptake compared to other East African countries (16). However, the country's contraception utilization is still mainly dominated by short-acting contraceptives, specifically injectables which account for 27% of contraceptive use among married women. According to the 2019 EDHS implants and IUDs only account for 9% and 2% of contraceptive use among married women, respectively (17).

Numerous studies in both developed and developing countries have examined factors influencing the low uptake of LARC. However, most focus on comparing LARC users with non-users, including those using short-acting or permanent methods. Age is a key factor, with older women more likely to choose LARC or permanent methods due to a desire to limit childbirth (18). Similarly, married women have higher LARC use, possibly due to a preference for delayed childbearing or provider biases favoring married clients (19, 20).

Religious affiliation also influences LARC use, with studies showing that followers of Orthodox and Protestant Christianity are more likely to use LARC, while Muslims are less likely to do so (21, 22). Additionally, LARC may appeal to women with limited household decision-making power, as it requires fewer healthcare visits and offers long-term effectiveness. In Ethiopia, limited research has explored the link between women's empowerment and LARC use, but available studies suggest a positive association measured using decision-making ability and LARC uptake (23, 24). Geographic location is another critical factor. Rural women often face barriers such as limited access to healthcare facilities and family planning information. While the introduction of Health Extension Workers (HEWs) has improved family planning uptake in Ethiopia (25), distance to healthcare services remains a significant obstacle to LARC use in rural areas (26, 27). Evidence of the impact of education level on the utilization of modern contraceptives is inconclusive (22, 28).

However, much of the existing literature focuses on identifying factors associated with the uptake of LARC compared to the remaining population (i.e., non-contraceptive users, short-acting, and permanent contraceptive users). From the intervention and policy perceptive, it is helpful to identify and understand the factors associated with low LARC uptake compared with each of these subgroups. Therefore, this study aimed to assess barriers and facilitators of LARC uptake compared to non-contraceptive users, traditional method users, and short-acting contraceptive users in Ethiopia. Identifying these factors will be important to tailor interventions and policies aiming to increase the uptake of LARC to target specific population groups. In addition, most users who utilize LARC in Ethiopia use implants over IUDs (17). This is despite both implants and IUDs having similar effectiveness at pregnancy prevention, and IUDs having the added advantage of being effective for a much more extended time (3 years vs. 10 years). Thus, this study further assessed factors contributing to the low uptake of IUDs among LARC users, which can help inform efforts to increase the utilization of this already available resource (IUDs).

## Methods

### Setting & participants

Ethiopia has nine regions [Amhara, Afar, Benishangul-Gumuz, Gambella, Harari, Oromia, Somali, Southern Nations, Nationalities and Peoples (SNNP), and Tigray] and two city administrations (Addis Ababa and Dire-Dawa). These 11 geographies are further divided into 74 zones. Data from the 2019 Performance Monitoring for Action Ethiopia (PMA Ethiopia) survey were used for this project. PMA Ethiopia further divided the 74 zones into 265 Enumeration areas (EAs). PMA Ethiopia is a project launched in 2014 and implemented in collaboration with Addis Ababa University, Johns Hopkins University, and the Ethiopian Federal Ministry of Health. It conducts a nationally representative survey annually measuring key reproductive, maternal, and child health indicators including modern contraceptive prevalence, reproductive empowerment, fertility intention, and health facility readiness and quality of care (29). PMA Ethiopia received ethical approval from Addis Ababa University, College of Health, and the Johns Hopkins University Bloomberg School of Public Health (JHSPH) Institutional Review Boards.

A cross-section of 35 households was randomly selected from within each EA, which were selected through a probability proportional to size sampling strategy. The household questionnaire was used to collect data on age, sex, and marital status of all usual members of the household or visitors who slept in the household the night before. All women aged 15–49 years old in the selected households were eligible for the cross-sectional survey. Once identified, they were approached for consent to participate in the individual female questionnaire from the household list. The women's questionnaire was used to measure indicators relevant to all women, such as family planning use, female empowerment, and reproductive decision-making and fertility intentions at that time point. Data collection was conducted between September and December 2019. A total of 8,837 women (98.5% of those selected) completed the cross-sectional survey (29, 30).

### Research design

#### Variables

##### Dependent variable

The primary outcome variable was the type of contraceptive method used by women aged 15–49. The type of contraceptive method used is measured based on two questions on PMA Ethiopia 2019. The first question asked, “Are you/your partner currently doing something or using any method to delay or avoid getting pregnant?” with “Yes” and “No” response options. Respondents who responded “Yes” were then asked, “Which method or methods are you using?” with 13 response options. For the purpose of this study, a nominal variable with four groups was created based on the response to the two questions:

1. Non-users: respondents responded “No” for the first question,
2. Traditional and barrier method users: respondents reported using Standard Days/Cycle Beads, lactational amenorrhea, rhythm method, withdrawal, male condoms, and female condoms as a contraceptive method.
3. Short-term contraceptive methods: respondents reported using injectables, pills, and emergency contraception as a contraceptive method.
4. LARC methods: respondents reported using Implants and IUDs as contraceptive methods.

If there were multiple responses to this contraceptive method question, these responses were excluded (*n* = 80) except when the multiple responses are condoms and another type of contraceptive method (*n* = 10). In this case, these responses were grouped to the contraceptive method mentioned other than condoms assuming the respondents are using condoms as prevention from STIs. Participants who reported using female and male sterilization (*n* = 15) were excluded from this study because, consistent with the national trend, only a few participants reported using these contraceptive methods (<1%). After excluding missing observations (*n* = 540), the final analytical sample was 8,182.

The sub-aim focused on women in the fourth group – LARC method users. For the sub-aim, the dependent variable was a binary variable of the type of LARC used by women. The two response options (Implant and IUDs) from the question “Which method or methods are you using?” were used to create this variable.

##### Independent variables

The selection of independent variables was guided by the Andersen Behavioral Model of Health Services, a widely used framework for assessing healthcare utilization and its determinants. This model has been extensively applied to evaluate health services, including reproductive health services in sub-Saharan Africa (31–33). It suggests that environmental factors (place of living) and individual characteristics (predisposing, enabling/impeding, and need factors) combine to influence health behavior, such as contraceptive use (34, 35). According to the Andersen Behavioral Model, predisposing factors influence an individual's likelihood of using health services (e.g., age, gender, residence type), enabling factors either facilitate or hinder access (e.g., health policies, wealth index, healthcare costs, number of providers), and need factors include both perceived needs (e.g., desire to delay childbirth) and evaluated needs (e.g., unmet family planning needs based on professional assessment) (34, 35).

**Figure 1:**
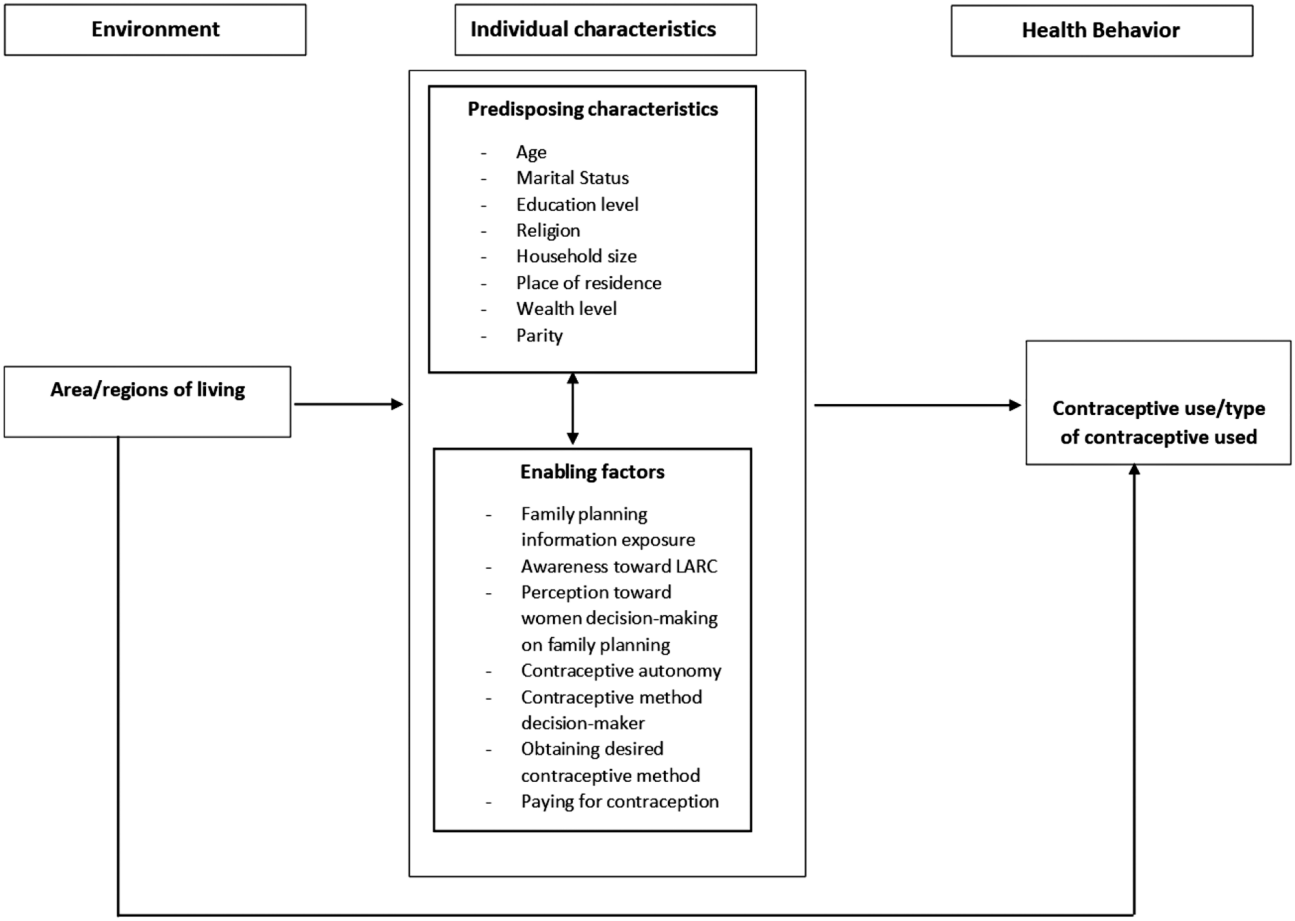
Conceptual framework for the study, adapted and revised from Changing the U.S. health care system: Key issues in health services policy and management, fourth edition (35).

In this study, as illustrated in Figure 1, independent variables across the three comparisons were categorized into individual-level predisposing and enabling factors. An environmental factor (area of residence) was also included in the multilevel model. Due to data limitations, need factors were not included.

##### LARC vs. non-contraceptive users

Predisposing individual-level characteristics included age (15–24, 25–34, or 35–49 years), marital status (married vs. not married), education level (no formal education, primary education only, or secondary and higher education level), religion (Orthodox, Muslim, Protestant or Other), place of residence (rural vs. urban), household size (less than four members vs. 4 or more members), wealth level (lowest quintile, low quintile, middle quintile, higher quintile, and highest quintile) and parity [nulliparous (no children) vs. multiparous (1 and more children)].

The enabling individual level characteristics included family planning information exposure, whether the participant agrees that women should be decision makers about family planning, and contraceptive autonomy. Family planning information exposure was assessed based on whether participants got information about family planning on radio, television, newspaper/magazine, through text message, or social media. Contraceptive autonomy is assessed based on the composite score for five 5-point Likert scale (strongly disagree to strongly agree) contraceptive autonomy questions from PMA Ethiopia. The composite score was computed by calculating the average score for each individual leading to a score range of 1–5. These five items were:

1. If I use family planning, my husband/partner may seek another sexual partner.
2. If I use family planning, I may have trouble getting pregnant the next time I want to.
3. If I use family planning, there could be/will be conflict in my relationship/marriage.
4. If I use family planning, my children may not be born normal.
5. If I use family planning, my body may experience side effects that will disrupt my relations with my husband/partner.

We used the reverse coded variables to create a composite score as a continuous variable; the Cronbach alpha for the five items was 0.76 (36, 37).

##### LARC vs. traditional and barrier methods

Similar predisposing and enabling individual factors were used in the comparison between LARC and non-contraceptive users. Due to the small sample size in the response option for the five-group variable, the wealth group variable is recoded into binary. The response options lowest, lower, and middle quintiles were classified as low wealth, while the response options higher and highest quintiles were classified as high wealth.

##### LARC vs. short-acting methods

The same predisposing individual-level characteristics as comparing the LARC vs. non-contraceptive user were used. The following enabling individual-level characteristics, in addition to the enabling characteristics above, were also included: who made the final decision on the contraceptive method woman used, whether the participant received the contraceptive method she desired, and whether the participant paid for the contraceptive method she received were included. These factors were only asked for individuals who received modern contraceptive methods therefore they were not included in the comparison with non-users and traditional and barrier methods.

##### Implants vs. IUDs

Due to the sample size for the sub-aim analysis (*n* = 734), some of the variables have small counts. Therefore, the predisposing individual-level characteristics included were age (younger than 35 years or 35 and older), marital status (married vs. not married), education level, religion (Orthodox or Other), place of residence, household size, and wealth level [low wealth group or high wealth group (which includes highest quintile and higher quintile)]. The enabling individual level characteristics included family planning information exposure contraceptive autonomy, who made the final decision on the contraceptive method the women use (not women or women/shared decision making), whether the participant agrees that women should be decision makers about family planning, whether the participant received the contraceptive method she desired and whether the participant paid for the contraceptive method she received. The full survey questionnaire used by PMA Ethiopia is available in Supplementary File 1.

#### Analysis

We started with weighted descriptive statistics examining the distribution of the independent variables for outcome by type of contraceptive used and type of LARC used.

Due to the nested hierarchy nature of PMA Ethiopia data, we used a multilevel model to assess factors associated with LARC uptake compared to the other three groups (38). Multilevel logistic regression models are used over a single multilevel multinomial regression model for two reasons; first, the possible factors associated with LARC uptake compared to non-users, traditional/barrier methods, and short-acting methods are different. While provider and health services-related factors could affect the choice between LARC and short-acting methods, these factors will not affect non-users. Second, running a separate multilevel logistic regression model is a more conservative approach than multilevel multinomial regression models and will provide more robust findings (39).

We conducted multilevel analyses using two types of clusters: regions and EAs. Based on Akaike Information Criterion (AIC) and Bayesian Information Criterion (BIC) model diagnostics, the models using EAs were the better fit. Therefore, the main analyses were performed with EAs as the cluster-level random effect. Before developing the multilevel logistic regression models, we assessed for correlation of errors for LARC uptake compared to non-LARC use by computing the analysis of variance estimate of the intraclass correlation coefficient (ICC) using the one-way ANOVA method. The ICC was 0.13, which means 13% of the variance in LARC uptake is due to clustering (EAs). There are no hard rules, but ICC of 0.10 or greater has been considered high enough that a multilevel model should be used (40).

For the assessment of factors associated with the use of IUDs compared to implants, a weighted logistic regression model was used due to the small total sample size (*n* = 743) and small sample size in the IUD group (*n* = 63). Because there was little variation among EAs, the multilevel logistic regression model failed to converge.

For all the analyses, a two-sided *p*-value less than 0.05 indicated statistical significance. The data cleaning and analyses were carried out in R statistical programming software (version 4.1.2).

## Results

### Participant characteristics

**Figure 2:**
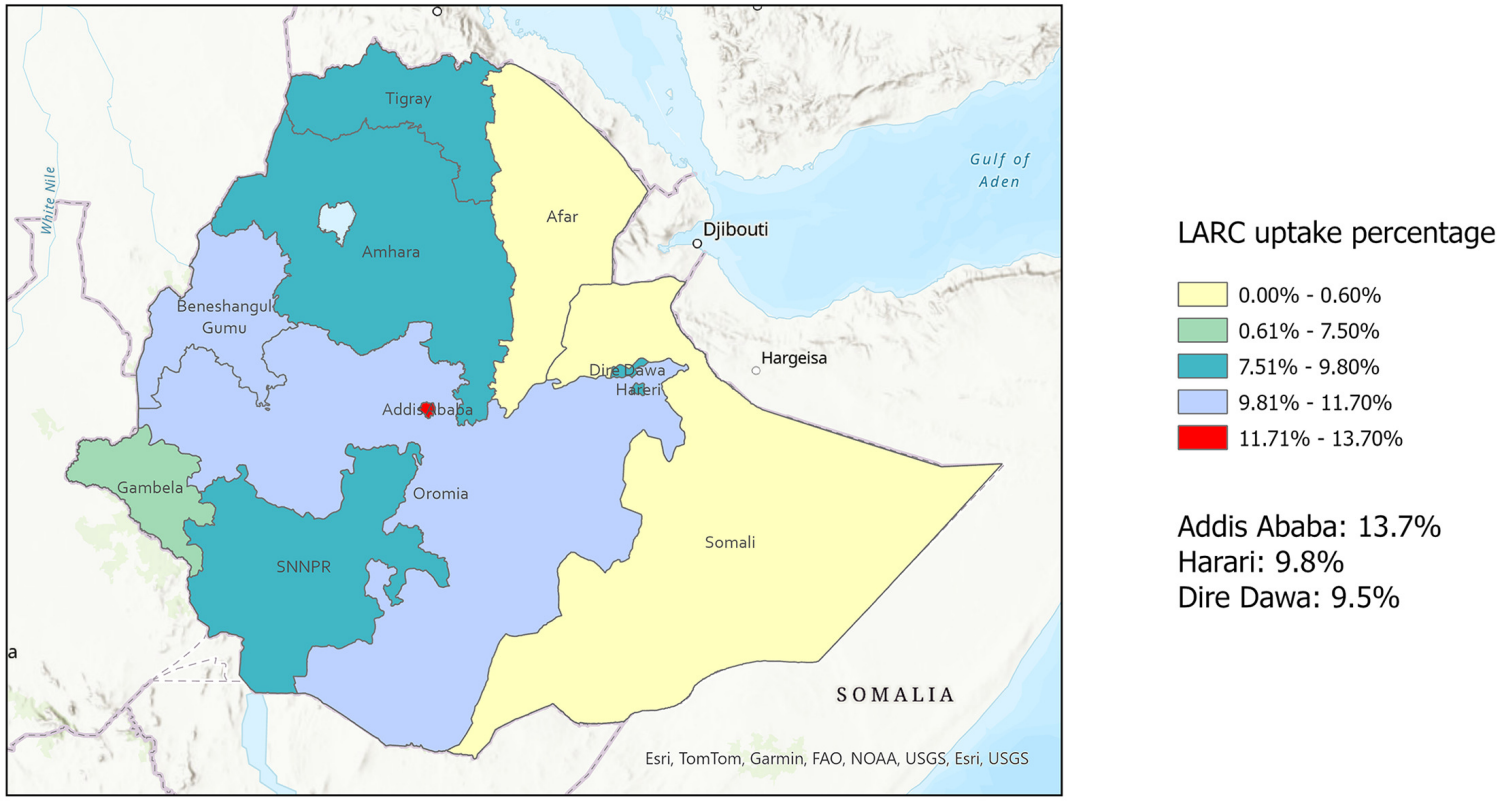
LARC uptake percentage in each region in Ethiopia, PMA Ethiopia survey (2019).

Less than one-third (29.3%) of the participants reported using contraception, with only 9.7% reporting using LARC as a contraceptive method. Looking at the weighted proportion of population who use LARC in each region, Addis Ababa has the highest proportion followed by the Benishangul-Gumuz region. The lowest were from Afar and Somali regions (Figure 2). The average age of LARC users was 29.45 (±0.32) which was the highest of all groups except traditional/barrier method users. Most of the LARC users were married, from households with 4 or more members, lived in rural areas, were Orthodox Christians, and multiparous (Table 1). Almost 89% of the participants reported they are aware of LARC. The proportion of non-contraceptive users who were aware of LARC was 87.7% compared to almost 100% among LARC users and 97.5% among short-acting method users.

**Table 1:** Weighted sample characteristics for contraceptive usage and type of contraceptive used: PMA Ethiopia survey (2019).

Variables	LARC	Non-user	Short acting	Traditional/barrier	Total
	*N* = 743 (9.67%)	*N* = 5,972 (70.97%)	*N* = 1,310 (17.57%)	*N* = 157 (1.75%)	(*N* = 8,182)
Age					
Mean (SD)	29.45 (0.32)	27.84 (0.16)	28.46 (0.24)	29.77 (0.73)	28.1 (0.13)
Age category					
15–24 years	189 (25.7%)	2,665 (44.5%)	413 (32.9%)	38 (27.8%)	3,305 (40.4%)
25–34 years	358 (46.9%)	1,581 (25.0%)	598 (42.7%)	68 (43.9%)	2,605 (31.8%)
35–49 years	196 (27.4%)	1,726 (30.5%)	299 (24.5%)	51 (28.3%)	2,272 (27.8%)
Household size					
Four and higher	543 (73.8%)	4,361 (76.1%)	869 (67.0%)	109 (72.5%)	5,882 (71.9%)
Less than 4	200 (26.2%)	1,611 (23.9%)	441 (33.0%)	48 (27.5%)	2,300 (28.1%)
Place of residence					
Urban	340 (34.8%)	2,562 (32.3%)	607 (36.6%)	100 (47.1%)	3,609 (44.1%)
Rural	403 (65.2%)	3,410 (67.7%)	703 (63.4%)	57 (52.9%)	4,573 (55.9%)
Wealth quantile					
Lowest quintile	97 (17.0%)	1,046 (20.7%)	145 (13.9%)	5 (4.8%)	1,293 (15.8%)
Lower quintile	112 (18.3%)	978 (19.3%)	195 (18.3%)	9 (8.4%)	1,294 (15.8%)
Middle quintile	124 (20.7%)	944 (18.8%)	237 (21.6%)	13 (11.9%)	1,318 (16.1%)
Higher quintile	143 (18.1%)	1,130 (19.6%)	281 (20.9%)	37 (28.9%)	1,591 (19.4%)
Highest quintile	267 (26.0%)	1,873 (21.6%)	452 (25.3%)	92 (46.0%)	2,684 (32.8%)
Missing	0 (0%)	1 (0.0%)	0 (0%)	1 (0.6%)	2 (0.0%)
Marital status					
Married	677 (92.2%)	2,959 (51.4%)	1,171 (91.2%)	128 (81.0%)	4,935 (60.3%)
Single	66 (7.8%)	3,013 (48.6%)	139 (8.8%)	29 (19.0%)	3,247 (39.7%)
Religion					
Muslim	114 (16.9%)	1,856 (31.6%)	204 (17.2%)	34 (20.1%)	2,208 (27.0%)
Orthodox	426 (54.2%)	2,838 (44.8%)	783 (57.1%)	73 (37.0%)	4,120 (50.4%)
Protestant	184 (26.3%)	1,155 (21.9%)	300 (23.5%)	48 (41.6%)	1,687 (20.6%)
Other	19 (2.6%)	121 (1.6%)	23 (2.2%)	2 (1.3%)	165 (2.0%)
Missing	0 (0%)	2 (0.0%)	0 (0%)	0 (0%)	2 (0.0%)
Education level					
Never attended	234 (36.9%)	2,075 (37.7%)	418 (36.2%)	28 (20.7%)	2,755 (33.7%)
Primary education	293 (40.6%)	2,036 (35.9%)	500 (39.1%)	40 (26.8%)	2,869 (35.1%)
Secondary and higher education	216 (22.5%)	1,859 (26.4%)	392 (24.7%)	89 (52.5%)	2,556 (31.2%)
Missing	0 (0%)	2 (0.0%)	0 (0%)	0 (0%)	2 (0.0%)
Parity					
Multiparous	679 (92.2%)	3,352 (57.8%)	1,132 (87.5%)	124 (75.6%)	5,287 (64.6%)
Nulliparous	64 (7.8%)	2,617 (42.2%)	177 (12.5%)	33 (24.4%)	2,891 (35.3%)
Missing	0 (0%)	3 (0.1%)	1 (0.1%)	0 (0%)	4 (0.0%)
Awareness of LARC					
No	3 (0.6%)	867 (12.1%)	39 (2.5%)	1 (1.3%)	910 (11.1%)
Yes	740 (99.4%)	5,099 (87.8%)	1,271 (97.5%)	156 (98.7%)	7,266 (88.8%)
Missing	0 (0%)	6 (0.1%)	0 (0%)	0 (0%)	6 (0.1%)
Family planning information exposure					
No	370 (56.8%)	3,291 (60.4%)	722 (59.9%)	59 (45.9%)	4,442 (54.3%)
Yes	370 (43.2%)	2,653 (39.2%)	584 (39.6%)	97 (53.4%)	3,704 (45.3%)
Missing	3 (0.1%)	28 (0.4%)	4 (0.5%)	1 (0.7%)	36 (0.4%)
Paid for the method you received?^a^					
No	668 (91.8%)	111 (2.0%)	897 (72.9%)	27 (15.4%)	1,703 (20.8%)
Yes	64 (6.7%)	11 (0.2%)	389 (25.9%)	7 (4.7%)	471 (5.8%)
Missing	11 (1.5%)	5,867 (97.9%)	24 (1.2%)	123 (80.0%)	6,008 (73.4%)
Obtained desired family planning^a^					
No	123 (17.7%)	0 (0%)	152 (11.3%)	2 (1.9%)	277 (3.4%)
Yes	619 (82.3%)	0 (0%)	1,126 (87.0%)	10 (7.0%)	1,755 (21.4%)
Missing	1 (0.1%)	5,989 (100%)	32 (1.7%)	145 (91.2%)	6,150 (75.2%)
Women are family planning option deciders					
No	463 (66.0%)	3,601 (60.3%)	821 (63.9%)	107 (76.3%)	4,992 (61.0%)
Yes	279 (34.0%)	2,332 (39.2%)	488 (36.1%)	50 (23.7%)	3,149 (38.5%)
Missing	1 (0.1%)	39 (0.5%)	1 (0.1%)	0 (0%)	41 (0.5%)
Who made the final decision about the method you use?^a^					
Partner	31 (4.2%)	0 (0%)	83 (6.4%)	0 (0%)	114 (1.4%)
Provider	58 (7.8%)	0 (0%)	25 (1.9%)	0 (0%)	83 (1.0%)
Women or shared decision making	651 (87.6%)	0 (0%)	1,171 (90.6%)	12 (7.6%)	1,834 (22.4%)
Missing	3 (0.4%)	5,989 (100%)	31 (1.1%)	145 (92.4%)	6,151 (75.2%)
Contraceptive autonomy					
Mean (SD)	4.00 (0.03)	3.61 (0.01)	3.93 (0.02)	3.92 (0.09)	3.71 (0.818)
Missing	6 (0.8%)	160 (2.7%)	5 (0.4%)	0 (0%)	171 (2.1%)

^a^ Asked for participants who received modern contraceptive methods (barrier, short-acting, or LARC).

Only 39% of all participants agreed women should be the ones to decide about family planning. Tigray region had the largest proportion of women who agreed women should be decision makers on family planning (74.5%), whereas Harari region had the smallest proportion (15.5%). The capital city Addis Ababa had also one of the lowest proportion of participants who agreed women should be decision makers on family planning (26.6%). Among women who received modern contraceptive methods, 90% of the women reported they made the final decision on the method they received by themselves or in collaboration with their partner or provider. This proportion is lower among LARC users (88%), with 8% reporting their provider made the final decision. There were additional differences between LARC users and the other groups by other individual characteristics (Table 1).

### Factors associated with LARC uptake compared to non-contraceptive use

From the predisposing individual-level characteristics age, religion, wealth, and parity were statistically significantly associated with LARC use compared to non-contraceptive use. The odds of LARC use were lower for participants who are in the older age group 35–49 years (aOR = 0.38, 95% CI 0.28–0.51 relative to 15–24 years), Muslims (aOR = 0.31, 95% CI 0.22–0.43 relative to Orthodox Christians), single (aOR = 0.13, 0.10–0.19), in lowest (aOR = 0.44, 95% CI 0.26–0.74 relative to highest quintile) and lower (aOR = 0.50, 95% CI 0.31–0.81 relative to highest quintile) wealth quintiles, and nulliparous (aOR = 0.20, 95% CI 0.14–0.29). Among the enabling individual-level characteristics, participants with higher contraceptive autonomy scores were more likely to be LARC users (aOR = 1.92, 95% CI 1.67–2.22) (Table 2).

**Table 2:** Odds ratios for LARC use compared to non-contraceptive use from multilevel logistic regression, PMA Ethiopia survey (2019).

Predictors	LARC use		
	aORs	95% CI	*p*-value
Age (Ref: 15–24 years)			
25–34 years	0.89	0.69–1.16	0.381
35–49 years	0.38	0.28–0.51	<0.001
Religion (Ref: Orthodox)			
Muslim	0.31	0.22–0.43	<0.001
Protestant	0.92	0.68–1.24	0.567
Other	1.19	0.61–2.35	0.606
Marital status (Ref: Married/living together)			
Single	0.13	0.10–0.19	<0.001
Education level (Ref: No formal education)			
Primary education	1.3	1.01–1.66	0.041
Secondary and higher education	1.25	0.90–1.74	0.176
Household size (Ref: 4 and more members)			
Less than 4	1.04	0.82–1.30	0.768
Parity (ref: Multiparous)			
Nulliparous	0.2	0.14–0.29	<0.001
Place of residence (Ref: Urban)			
Rural	1.23	0.79–1.91	0.363
Wealth quintile (Ref: Highest quintile)			
Lowest quintile	0.44	0.26–0.74	0.002
Lower quintile	0.5	0.31–0.81	0.005
Middle quintile	0.63	0.39–1.00	0.051
Higher quintile	0.8	0.55–1.15	0.219
Family planning Information exposure (Ref: Yes)			
No	0.89	0.72–1.11	0.304
Women should be the ones to decide about FP (Ref: No)			
Yes	1.08	0.87–1.34	0.483
Contraceptive autonomy	1.92	1.67–2.22	<0.001
Random Effects			
σ^2^	3.29		
τ_00__EA_	0.95		
ICC	0.22		
N_EA_	265		
Observations	6,510		
Marginal R^2^/Conditional R^2^	0.384/0.521		
Model summary			
AIC	3,477.4		
BIC	3,613.0		
Log likelihood	−1,718.7		

aOR, adjusted odds ratio; CI, confidence interval; EA, enumeration area; ICC, inter class correlation; Ref, reference group.

### Factors associated with LARC uptake compared to traditional/barrier contraceptive method use

From the predisposing individual-level characteristics age, religion, education level, wealth, and parity were statistically significantly associated with LARC use compared to traditional and barrier method use. The odds of LARC use were lower for participants who were in the age group 35–49 years (aOR = 0.23, 95% CI 0.10–0.53 relative to 15–24 years), Muslims (aOR = 0.32, 95% CI 0.15–0.68) and Protestant (aOR = 0.45, 95% CI 0.24–0.85 relative to Orthodox Christians), who had secondary and higher education level (aOR = 0.23, 95% CI 0.10–0.54 relative no formal education), who were in high wealth group (aOR = 0.37, 95% CI 0.17–0.82), and nulliparous women (aOR = 0.24, 95% CI 0.10–0.60). Furthermore, from the enabling individual-level characteristics, contraceptive autonomy was a statistically significant factor. Women with higher contraceptive autonomy scores (aOR = 1.93, 95% CI 1.35–2.76) were more likely to use LARC as a contraceptive method (Table 3).

**Table 3:** Odds ratios for LARC use compared to traditional/barrier method use from multilevel logistic regression, PMA Ethiopia survey (2019).

Predictors	LARC use		
	aORs	95% CI	*p*-value
Age (Ref: 15–24 years)			
25–34 years	0.57	0.28–1.16	0.122
35–49 years	0.23	0.10–0.53	<0.001
Religion (Ref: Orthodox)			
Muslim	0.32	0.15–0.68	0.003
Protestant	0.45	0.24–0.85	0.014
Other	1.01	0.17–6.14	0.993
Marital status (Ref: Married/living together)			
Single	0.54	0.24–1.18	0.123
Education level (Ref: No formal education)			
Primary education	0.71	0.34–1.46	0.351
Secondary and higher education	0.23	0.10–0.54	0.001
Household size (Ref: 4 and more members)			
Less than 4	1.08	0.61–1.93	0.788
Parity (ref: Multiparous)			
Nulliparous	0.24	0.10–0.60	0.002
Place of residence (Ref: Urban)			
Rural	1.34	0.58–3.10	0.487
Wealth group (Ref: Low wealth)			
High wealth	0.37	0.17–0.82	0.014
Family planning Information exposure (Ref: Yes)			
No	0.83	0.49–1.42	0.497
Women should be the ones to decide about FP (Ref: No)			
Yes	1.65	0.94–2.90	0.084
Contraceptive autonomy	1.93	1.35–2.76	<0.001
Random effects			
σ^2^	3.29		
τ_00__EA_	1.98		
ICC	0.38		
N_EA_	218		
Observations	890		
Marginal R^2^/Conditional R^2^	0.245/0.529		

aOR, adjusted odds ratio; CI, confidence interval; EA, enumeration area; ICC, inter class correlation; Ref, reference group.

### Factor associated with LARC update compared to short-acting contraceptive method use

From the predisposing individual-level characteristics, household size was statistically significantly associated with LARC use compared to short-acting method use. The odds of LARC use were lower for participants with less than 4 household members (aOR = 0.74, 95% CI 0.56–0.98). Furthermore, from the enabling individual level characteristics family planning information exposure, paying for contraceptive method, who made the final decision on contraceptive method received, and contraceptive autonomy were statistically significant factors. The odds of LARC use were lower for participants who had no exposure to family planning information (aOR = 0.72, 95% CI 0.56–0.93), who paid for the contraceptive method they received (aOR = 0.145, 95% CI 0.10–0.21), and among those who made contraceptive method choices by themselves (aOR = 0.26, 95% CI 0.14–0.46) and those whose partner decided their contraceptive method (aOR = 0.19, 95% CI 0.09 −0.41) relative to whose healthcare providers decided their contraceptive method. In addition, women with a higher score of contraceptive autonomy were more likely to use LARC (aOR = 1.25, 95% CI 1.05–1.49). Despite not being statistically significant, a borderline statistically significant finding was also observed for whether the woman obtained the method she desired. The point estimate showed women who obtained their desired method were less likely to use LARC than short-acting methods (aOR = 0.74, 95% CI 0.53–1.03) (Table 4).

**Table 4:** Odds ratios for LARC use compared to short-acting contraceptives method use from multilevel logistic regression, PMA Ethiopia survey (2019).

Predictors	LARC use		
	aORs	95% CI	*p*-value
Age (Ref: 15–24 years)			
25–34 years	1.1	0.81–1.49	0.551
35–49 years	1.19	0.82–1.73	0.359
Religion (Ref: Orthodox)			
Muslim	1.01	0.70–1.45	0.971
Protestant	1.16	0.83–1.61	0.383
Other	0.92	0.42–2.02	0.841
Marital status (Ref: Married/living together)			
Single	1.45	0.95–2.22	0.088
Education level (Ref: No formal education)			
Primary education	1.05	0.78–1.41	0.749
Secondary and higher education	1.11	0.76–1.62	0.596
Household size (Ref: 4 and more members)			
Less than 4	0.74	0.56–0.98	0.039
Parity (ref: Multiparous)			
Nulliparous	1.13	0.72–1.75	0.597
Place of residence (Ref: Urban)			
Rural	0.88	0.55–1.40	0.583
Wealth quintile (Ref: Highest quintile)			
Lowest quintile	0.82	0.46–1.47	0.504
Lower quintile	0.76	0.44–1.32	0.33
Middle quintile	0.76	0.45–1.29	0.307
Higher quintile	0.76	0.50–1.14	0.183
Family planning Information exposure (Ref: Yes)			
No	0.72	0.56–0.93	0.01
Who made the final decision about the method you received? (Ref: Provider)			
Partner	0.19	0.09–0.41	<0.001
Women	0.26	0.14–0.46	<0.001
Women should be the ones to decide about FP (Ref: No)			
Yes	0.91	0.71–1.16	0.441
Paid for the method received (Ref: No)			
Yes	0.15	0.10–0.21	<0.001
Obtained desired family planning method (Ref: No)			
Yes	0.74	0.53–1.03	0.076
Contraceptive autonomy	1.25	1.05–1.49	0.011
Random effects			
σ^2^	3.29		
τ_00__EA_	0.75		
ICC	0.18		
N _EA_	240		
Observations	1,984		
Marginal R^2^/Conditional R^2^	0.160/0.316		

aOR, adjusted odds ratio; CI, confidence interval; EA, enumeration area; ICC, inter class correlation; Ref, reference group.

### Factor associated with IUDs use compared to implants

The weighted proportion of IUD use among LARC users was 6.9%. Compared to implant users, IUD users were older, living in urban areas, in a high-wealth group, multiparous, and with lower contraceptive autonomy scores. There were also differences between Implant and IUD users by other individual characteristics (Table 5).

**Table 5:** Weighted sample characteristics for the type of LARC use: PMA Ethiopia survey (2019).

Variables	Implant	IUD	Total
	*N* = 680 (93.1%)	*N* = 63 (6.9%)	(*N* = 743)
Age			
Mean (SD)	29.15 (0.33)	33.47 (1.17)	29.45 (0.32)
Median [min, max]	28.0 [15.0, 49.0]	33.0 [20.0, 49.0]	28.0 [15.0, 49.0]
Age Category (two groups)			
15–34 years	508 (73.8%)	39 (56.2%)	547 (73.6%)
35–49 years	172 (26.2%)	24 (43.8%)	196 (26.4%)
Household size			
Four and higher	495 (73.2%)	48 (82.7%)	543 (73.1%)
Less than 4	185 (26.8%)	15 (17.3%)	200 (26.9%)
Place of residence			
Urban	293 (32.9%)	47 (60.7%)	340 (45.8%)
Rural	387 (67.1%)	16 (39.3%)	403 (54.2%)
Wealth binary			
High quantile	359 (42.2%)	51 (68.7%)	410 (55.2%)
Low quantile	321 (57.8%)	12 (31.3%)	333 (44.8%)
Marital status			
Married	623 (92.5%)	54 (87.6%)	677 (91.1%)
Single	57 (7.5%)	9 (12.4%)	66 (8.9%)
Religion binary			
Orthodox	388 (54.5%)	38 (49.5%)	426 (57.3%)
Other	292 (45.5%)	25 (50.5%)	317 (42.7%)
Education level			
Never attended	218 (37.3%)	16 (30.6%)	234 (31.5%)
Primary	274 (41.2%)	19 (33.0%)	293 (39.4%)
Secondary and higher	188 (21.5%)	28 (36.5%)	216 (29.1%)
Parity			
Multiparous	618 (91.8%)	61 (96.9%)	679 (91.4%)
Nulliparous	62 (8.2%)	2 (3.1%)	64 (8.6%)
Family planning information exposure			
No	349 (57.4%)	21 (48.3%)	370 (49.8%)
Yes	329 (42.6%)	41 (51.7%)	370 (49.8%)
Missing	2 (0.3%)	1 (1.6%)	3 (0.4%)
Women are family planning option deciders			
No	425 (66.0%)	38 (65.7%)	463 (62.3%)
Yes	255 (34.0%)	24 (34.3%)	279 (37.6%)
Missing	0 (0%)	1 (1.6%)	1 (0.1%)
Obtained desired family planning			
No	109 (17.1%)	14 (25.6%)	123 (16.6%)
Yes	571 (82.9%)	48 (74.4%)	619 (83.3%)
Missing	0 (0%)	1 (1.6%)	1 (0.1%)
Paid for the method received			
No	615 (93.2%)	53 (92.9%)	668 (89.9%)
Yes	57 (6.8%)	7 (7.1%)	64 (8.6%)
Missing	8 (1.2%)	3 (4.8%)	11 (1.5%)
Who made the final decision about the method you choose?			
Not women	78 (11.2%)	11 (21.4%)	89 (12.0%)
Women	600 (88.8%)	51 (78.6%)	651 (87.6%)
Missing	2 (0.3%)	1 (1.6%)	3 (0.4%)
Contraceptive autonomy			
Mean (SD)	4.05 (0.686)	3.81 (0.823)	4.03 (0.701)
Median [min, max]	4.00 [1.00, 5.00]	4.00 [1.20, 5.00]	4.00 [1.00, 5.00]
Missing	5 (0.7%)	1 (1.6%)	6 (0.8%)

Percentages in this table are survey-weighted.

From the predisposing individual-level characteristics age and place of residence were statistically significantly associated with IUD use compared to implant use. The odds of IUDs use were lower for women younger than 30 years (aOR = 0.34, 95% CI 0.15, 0.82) and women who lived in rural areas (aOR = 0.33, 95% CI 0.17, 0.65). None of the assessed enabling factors were statistically significant (Table 6).

**Table 6:** Odds ratios for IUDs use compared to implants use from weighted logistic regression, PMA Ethiopia survey (2019).

Predictors	aOR	95% CI
Age (Ref: 30–49 years)		
15–29 years	0.34**	0.15–0.82
Marital Status (Ref: Single)		
Married	0.42	0.16–1.09
Religion (Ref: Orthodox)		
Other	1.59	0.77–3.31
Education level (Ref: Secondary and higher education)		
No formal education	0.56	0.21–1.48
Primary education	0.59	0.25–1.39
Household size (Ref: 4 and more members)		
Less than 4	0.62	0.24–1.59
Family planning Information exposure (Ref: No)		
Yes	0.84	0.46–1.56
Place of residence (Ref: Urban)		
Rural	0.33***	0.17–0.65
Wealth level (Ref: High wealth group)		
Low wealth group	0.67	0.29–1.54
Who made the final decision about the method you got? (Ref: Not women)		
Women	0.43*	0.18–1.02
Women should be the ones to decide about FP (Ref: No)		
Yes	0.96	0.48–1.91
Obtained desired family planning (Ref: No)		
Yes	0.59	0.25–1.36
Paid for the method received (Ref: No)		
Yes	0.83	0.24–2.85
Contraceptive autonomy	0.60*	0.34–1.09

aOR, adjusted odds ratio; CI, confidence interval; Ref, reference group.

* *p* < 0.1.

** *p* < 0.05.

*** *p* < 0.01.

## Discussion

This study aimed to assess individual-level factors associated with LARC uptake using Andersen's Behavioral Model of Health Services Use. Using multilevel analysis, we assessed individual-level predisposing and enabling factors associated with LARC uptake compared to non-contraceptive use, traditional/barrier method use, and short-acting contraceptive method use. Age, marital status, parity, wealth index, religion, and contraceptive autonomy were all associated with LARC use compared to non-contraceptive use. Household size, contraceptive methods cost, family planning information exposure, contraceptive method decision maker, and contraceptive autonomy were all associated with LARC use compared to short-acting methods. In addition, as a sub-aim, we assessed individual-level predisposing and enabling factors associated with IUD use compared to Implant use. Age and place of residence were associated with IUD use compared to implant use.

Overall, only around 30% of the participants used some type of family planning method. In addition, short-acting methods continue to account for the majority of contraceptive use, comprising 60% of all users, with LARC being used by 33% of contraceptive users. These findings are in line with previous national studies from Ethiopia (2, 41). This shows that Ethiopia did not meet its target for LARC to constitute 50% of all modern contraception use by 2020 (42).

### Women's decision-making in family planning

The findings of this study showed the perception of women's decision-making in family planning requires more work. Only 39% of the participants agreed women should have decision-making power in family planning. The survey question used for this variable only asked about women's decision-making and not about shared decision making. This finding is similar to the finding from DHS 2016 which reported that only 30% of married women had independent decision-making power on whether or not to use any contraceptives (43). In contrast, the current study's finding on women's decision-making is lower than a finding from a small regional study from Tigray (44). The reason for this discrepancy could be the size of the study (nationwide sample vs. regional sample). In our study, three-fourths of women from the Tigray region agreed women should have decision-making power in family planning. The current study's finding also showed that the capital city Addis Ababa also had one of the lowest proportions of women who agreed on women's decision-making power in family planning. This shows there is a huge discrepancy between regions in perceptions of women's decision-making power on family planning, and even in big cities such as Addis Ababa the problem is widespread. The experience of the Tigray region can serve as a model for future efforts to improve women's family planning decision-making.

### Predisposing factors associated with LARC uptake

In the current study, age, religion, parity, and wealth index were found to be associated with LARC uptake compared to non-contraceptive use and traditional/barrier method use. Older women were less likely to use LARC. A similar finding is reported in a study from Ethiopia which found older age is negatively associated with the use of modern contraceptives (45). However, our finding is contrary to research that shows women in the age group 20–29 were the group most likely to use LARC (23) and others that reported no significant difference based on age (46). A possible explanation could be the difference in the measurement of age, outcome variable, and statistical methodology. Consistent with other national and regional studies (26, 46–50), we found that multiparous women are more likely to use LARC than nulliparous women. In addition, the current study also found married women are more likely to use LARC compared to single women. Moreover, as LARC methods are suitable for all age groups, parity, and marital status, it is important to make sure that LARC methods are offered to all eligible groups. Future studies are recommended to further investigate the role of providers in the LARC uptake difference based on age, marital status, and parity.

As a predisposing factor based on our theoretical framework, a higher wealth index was associated with the uptake of LARC compared to non-contraceptive use. Our finding shows that women in the lower and lowest wealth indices were more than 100% less likely to use LARC. This finding is in line with other regional and national studies from Ethiopia (23, 41, 50, 51) and sub-Saharan African countries (52). In contrast, women with high wealth index and women with secondary or higher education level were less likely to use LARC than traditional and barrier methods. Previous studies that assess LARC uptake compared to other modern contraceptive methods use reported women with high wealth were more likely to use LARC (23). However, evidence from the U.S. showed women with higher education level are more likely to use condoms as a contraceptive. A similar report showed there was no difference in LARC uptake based on education level (53). Further studies are needed to understand the association between high wealth index an higher education level with the use of traditional and barrier method when compared to LARC. Overall, wealth index and high education attainment are closely associated with the availability of resources, proximity to health facilities, and health literacy (54). These associations possibly explain the association between low wealth index and low LARC uptake when compared to non-contraceptive use. These findings imply that Ethiopia's family planning program will benefit from prioritizing targeted interventions such as increasing access to LARC and health education, to support the most underprivileged segments of the population.

Religion was also found to be associated with the use of LARC compared to non-contraceptive use and traditional/barrier method use. Our study shows that Muslim women are less likely to use LARC compared to Orthodox Christian women. In addition to Muslims, Protestant were also less likely to use LARC compared to traditional/barrier method use. These findings are consistent with previous national and regional studies from Ethiopia on LARC use and modern contraceptive which reported Muslims are less likely to use LARC and modern contraceptive methods (22, 28, 55–58). The studies from Ethiopia also showed that compared to Muslim women, Orthodox Christian women are more likely to use modern contraceptives (21, 56, 57). Studies from the U.S. showed Muslim women believe their religion permits the use of reversible contraceptive methods, and Muslim women have a similar or higher rate of contraceptive use compared to the general population (59, 60). These findings suggest that religious leaders of Islam in Ethiopia could play an important role in increasing the acceptability and uptake of LARC among their followers (61). Future studies are needed to explore the complex interplay between religion, wealth, culture, and contraceptive use. This study shows larger household size is associated with higher LARC uptake compared to short-acting contraceptive methods. Similar findings are reported in studies that assessed the association between the number of living children and LARC use. These studies showed women are more likely to use LARC as the number of living children increases (62). One possible explanation for these findings could be that as the number of household size increases there will be limited resources for more children leading to the use of LARC methods over short-acting methods to limit childbearing for a longer duration.

### Enabling factors associated with LARC uptake

This study shows contraceptive autonomy is positively associated with LARC uptake compared to non-contraceptive use, traditional/barrier method use, and short-acting method use. Contraceptive autonomy was measured using five questions that assess the perception of women toward their partner/husband's response to them using contraceptives and their perception toward the adverse effects of contraception. This finding is consistent with previous studies that found a link between women's empowerment and LARC use, though women's empowerment was measured differently in previous studies, and the comparisons were between LARC use and non-LARC use (23, 28). Moreover, similar findings are reported in studies from other African countries (63–65) and the U.S. where LARC users reported increased reproductive autonomy (66). Policies and interventions to increase women's empowerment and contraceptive autonomy must be included in efforts to increase LARC uptake.

Among short-acting and LARC users, the enabling factors family planning information exposure, cost of contraceptive methods, and who made the final decision of contraceptive method were found statistically significant. Exposure to family planning information through different media was associated with a higher uptake of LARC compared to short-acting contraceptive methods. This finding is in agreement with studies from Ethiopia (23, 41) and other sub-Saharan African countries (48). Overall, these finding shows the importance of access to family planning information. Misconceptions, misinformation, and misinterpreted side effects are some of the major barriers to LARC uptake, and increasing access to family planning information through community health workers (67) and mobile health interventions (68) could play a notable role in tackling these barriers.

Another enabling factor for LARC uptake was the cost of contraceptive methods. According to the current study, women were less likely to use LARC than short-acting methods when they had to pay for the contraceptive method. This is despite in Ethiopia most contraceptive methods are provided for free in public health facilities. In the current study, less than one-fourth of modern contraceptive method users reported that they paid for the contraceptive method they received. This finding is in line with studies from the U.S. that showed ones the cost barrier is removed women were more likely to choose LARC as a contraceptive method (69, 70). Health extension workers can play a key role in raising awareness about the free family planning services available, including LARC, at public health facilities.

Consistent with previous findings (41, 48, 71, 72), the current study also shows when the contraceptive method is chosen by health providers, women are more likely to receive LARC over short-acting compared to when women by themselves or through shared-decision making decide the contraceptive method they want to use. This finding is further supported by the current study's finding that when women obtain their desired contraceptive method, they are less likely to use LARC. According to qualitative studies from Ethiopia and other sub-Saharan African nations, women felt pressured to use LARC by healthcare professionals and said they were given LARC against their will (71, 73). The vigorous adoption of health policies that target to meet quotas for particular contraceptive techniques may have played a role in this (73). A system that offers incentives for health facilities and providers to fulfill pre-planned goals for the number of contraceptive methods they provide is part of Ethiopia's healthcare policy strategy. This might prompt healthcare professionals to provide LARCs to women even though they would prefer to use a different type of contraceptive method (73). Based on these findings, we should explore why women prefer short-acting contraceptive methods over LARC. Women's mistrust of utilizing LARC is partly due to misconceptions and myths regarding its side effects (71, 73). Further efforts are warranted to combat the misconceptions and myths about LARC. Overall, the WHO recommendations for family planning counseling and provision are generally based on respecting the client's family planning choice and enabling informed decision-making (74). The results of this study, when coupled with findings from earlier studies, indicate that while it is important to promote the use of LARC, we should be careful not to overpromote LARC (66, 75, 76), and the family planning service should be guided by the informed choice principle as established by the WHO (74).

### Factors associated with IUD use compared to implant use

In Ethiopia, the use of IUDs is still very low. According to our findings, only 7% of LARC users have an IUD. This is comparable to the report from EDHS 2016, where only 2% of currently married modern contraceptive users were using IUDs (2), and another multi-region study from Ethiopia found that only 5% of LARC users were using IUDs (77). Despite the Ethiopian government's efforts to increase IUD utilization (42), these findings indicate a lack of progress and the need for additional efforts to understand IUD hesitance and increase IUD utilization.

In this study, different individual-level factors were found to be associated with IUD use compared to Implants. As a predisposing factor based on our theoretical framework of Andersen's Behavioral Model of Health Services Use, age was associated with IUD use compared to Implants. Our finding shows older women are more likely to use IUDs compared to Implants. A mixed-method study conducted in Ethiopia found that the majority of women seeking to use IUDs were young women (77). This demonstrates that there is a demand for IUDs among young women, but there might be a lack of access to them. Furthermore, the current study shows rural women are less likely to use IUDs than Implants. The deployment of Health Extension Workers (HEWs) to rural areas increases the uptake of Implants (67). However, HEWs are not eligible to insert IUDs, which may explain why IUD use is lower in rural areas. A study conducted to evaluate the impact of the Ethiopian government's IUD initiative program using a before-after study design found that after the program was implemented, IUD use increased 14-fold, with rural women accounting for the majority of the demand (77). This suggests that increasing access through training HEWs to insert IUDs could play a role in increasing IUD uptake in rural areas. None of the enabling factors in this study were statistically significant, likely due to the small sample size. Further research with a larger sample is needed to better understand the lower uptake of IUDs compared to implants.

## Limitations

The findings in this study are subject to the following limitations. First, the PMA 2019 data were collected through a cross-sectional study design, thus, we cannot establish causal and temporal associations. In addition, recall bias and social desirability bias in the self-reported measurements of the type of contraceptive method used, decision-making and payment for contraceptive method used, and contraceptive autonomy cannot be ruled out (78). Secondly, we were not able to assess the need component of Andersen's Behavioral Model of Health Services Use because these factors were not available, or they were not complete on PMA 2019 Ethiopia survey. The need factors previously found to affect LARC uptake that we were not able to assess include intention to delay pregnancy (28), desire for more children (26, 46), and for how long women desire to delay pregnancy (23). Thirdly, we could not assess all predisposing and enabling factors based on our theoretical framework as they were not available in our survey. Examples of these factors include occupation and knowledge and perception toward LARC (23, 26, 72). Fourth, we could not include environmental and system-level factors, such as healthcare availability (27), which significantly impact LARC uptake. Finally, we were not able to use survey weighting on the multilevel model as the R statistical package does not have a survey weighting function for the multilevel model. However, we conducted a survey weight logistic regression and compared to our findings. The findings are mostly the same with few differences in significance.

## Conclusions

We identified predisposing and enabling factors associated with LARC uptake compared to non-contraceptive use, traditional and barrier methods, and short-acting methods. Our findings showed older, single, nulliparous, and Muslim women had lower LARC use than non-contraceptive and traditional/barrier method users. When compared to short-acting method use, low LARC use was associated with smaller household size and no exposure to family planning information. When compared to all other groups, contraceptive autonomy was associated with higher LARC uptake. Efforts are needed to increase LARC use among young, nulliparous, single, and Muslim women. More work is needed to improve women's family planning decision-making ability and providers’ respect for women's contraceptive method choice. While more research on women's disinclination to use LARC as a contraceptive method is needed, the findings from this study could be used to develop interventions and policies to improve LARC access and uptake in populations with low uptake. Furthermore, our findings could be used to advocate for increased access to IUDs in rural areas and among young women.

## Public health and policy implication

Based on the findings of this study—while acknowledging its limitations, including the cross-sectional design—public health policymakers should prioritize delivering family planning services guided by informed decision-making principles. The WHO's family planning services provision guide is built around informed decision-making (74). Furthermore, our findings showed that increasing women's contraceptive autonomy and decision-making would be more effective in increasing LARC uptake among women. We recommend that policymakers and other stakeholders working on increasing LARC uptake to develop effective family planning counseling training programs for family planning providers emphasizing informed decision-making and LARC eligibility, as well as increase women's exposure to family planning information via various forms such as radio, television, newspapers, and direct visits from community health workers.

## Data Availability

Publicly available datasets were analyzed in this study. This data can be found here: https://datalab.pmadata.org/dataset/doi%3A10349766hen-vd80.
