# Supplementary material for: Examining the low uptake of LARC in Ethiopia: an analysis of individual-level predisposing and enabling factors
Source: Front Glob Womens Health. 2025 Jun 6;6:1547891. doi: 10.3389/fgwh.2025.1547891 (PMC12179146; doi:10.3389/fgwh.2025.1547891)
Supplement: Supplementary file 1 [file Datasheet1.pdf]

PMA-Ethiopia 2019 Cross-Sectional Survey

Female Questionnaire

Ethiopia Female Questionnaire—Cross-Section	
WARNING: You have opened a female respondent questionnaire that is not linked to a household roster.	
A. Are you in the correct household?<br>*EA: ${EA} Structure #: ${structure} Household #: ${household}*	◯ Yes<br>◯ No
RETURN TO INTERVIEW THE CORRECT HOUSEHOLD.	
Your Name	
C. Is this your name?	◯ Yes<br>◯ No
D. Enter your name below.<br>*Please record your name*	
Current Date-Time:	Day:<br>Month:<br>Year:
E. Is this date and time correct?	◯ Yes<br>◯ No
F. Record the correct date and time.	Day:<br>Month:<br>Year:
	Location Detail - Not linked to a household roster.
Region:	◯ Afar<br>◯ Ethiopia Somali<br>◯ Benishangul Gumuz<br>◯ Gambela<br>◯ Hareri<br>◯ Dire Dawa Astedadar<br>◯ Tigray<br>◯ Amhara<br>◯ Oromiya<br>◯ Snnp<br>◯ Addis Ababa

Zone:	*ODK will populate a list of appropriate zones based on the selected region.*
District:	*ODK will populate a list of appropriate districts based on the selected zone.*
Locality:	*ODK will populate a list of appropriate localities based on the selected district. There may be only one choice.*
Enumeration area	*ODK will populate the appropriate EA.*
Structure number	
Household number	

	Location Detail
The following information is from the Household Questionnaire. Please review to make sure you are interviewing the correct respondent	
Region:	
Zone:	
District:	
Locality:	
Enumeration area	
Structure number	
Household number	
G. Is the above information correct?	◯ Yes<br>◯ No

Go to the right household or update the household Questionnaire if needed.	
I. CHECK: You should be attempting to interview ${firstname}. Is that correct?<br>*If misspelled, select “Yes” here and update the name in question "P” If this is the wrong person, you have two options: (1) exit and ignore changes to this form. Open the correct form. Or (2) find and interview the person whose name appears above*	◯ Yes<br>◯ No
J. Is the respondent present and available to be interviewed today?	◯ Yes<br>◯ No
K. How well acquainted are you with the respondent?	◯ Very well acquainted<br>◯ Well acquainted<br>◯ Not well acquainted<br>◯ Not acquainted

CROSS-SECTION INFORMED CONSENT<br>*Find the woman between the ages of 15-49 associated with this Female Questionnaire. The interview must have auditory privacy. Read the following greeting:*	
Hello. My name is ______________________ and I am working for the Addis Ababa University, and Federal Ministry of Health. We are conducting a local survey that asks women about various reproductive health issues, including family planning and pregnancy using a smartphone. The survey helps monitor the state of public health and questions will be used for research purposes. We would very much appreciate your participation in this survey. This information will help us inform the government to better plan health services. The survey usually takes between 30 and 40 minutes to complete. Whatever information you provide will be kept strictly confidential. Your data will not be linked to your identity when conducting analyses, presenting results, or sharing data.<br>Participation in this survey is entirely voluntary. If we should come to any question you don't want to answer, just let me know and I will go on to the next question; or you can stop the interview at any time. However, we hope that you will participate in this survey since your views are important. If you choose to participate in the survey, you will receive 25 birr airtime credit.<br>If you have any questions about the study and your rights as a research participant, you may ask me now or you may also contact the principal investigators of the study, Solomon Shiferaw (251-911-406845) or Assefa Seme (251-911-228193). For any ethical issues, please call Dr. Solomon Abay, the IRB chairperson (011-251 941 222169) at the Addis Ababa University, College of Health Sciences.<br>At this time, do you want to ask me anything about the survey?	
M. May I begin the interview now?	◯ Yes<br>◯ No
O. Interviewer's name: ${your_name}<br>*Mark your name as a witness to the consent process.*	◯
O. Interviewer's name<br>Please record your name as a witness to the consent process. You previously entered "${name_typed}."	
P. Respondent's first name<br>*You may correct the spelling here if it is not correct, but you must be interviewing the person whose name appears below.*	

Section 1 – Respondent's Background, Marital Status, and Household Characteristics

Now I would like to ask about your background and socioeconomic conditions.

FFQ001. In what month and year were you born? The age in the household roster is ${age}.<br>*If respondent knows the year, but not month select the 'Does not know month' checkbox. Select the 'Do not know' checkbox for month and '2030' for year to indicate 'No Response'.*	
Month and Year	Month:<br>Year:
Check here if respondent does not know month.	☐
FFQ002. How old were you at your last birthday?	
FFQ003a. Are you currently married or living together with a man as if married?<br>*Probe: If no, ask whether the respondent is divorced, separated, widowed, or never in union/never married*	◯ Yes, currently married<br>◯ Yes, living with a man<br>◯ Divorced / separated<br>◯ Not currently in union: widow<br>◯ No, never in union<br>◯ No response
FFQ003b. Have you been married or lived with a man only once or more than once?	◯ Only once<br>◯ More than once<br>◯ No response
FFQ004a. In what month and year did you start living with your FIRST husband / partner?<br>*If respondent knows the year, but not month select the 'Does not know month' checkbox. Select the 'Do not know' checkbox for month and '2030' for year to indicate 'No Response'.*	
Month and Year	Month:<br>Year:
Check here if respondent does not know month.	☐
FFQ004b. CHECK: Based on the response you entered in FFQ004a, the respondent was possibly 15 years old or younger at the time of her first marriage. Did you enter FFQ004a correctly?	◯ Yes<br>◯ No
FFQ005a. Now I would like to ask about when you started living with your CURRENT or MOST RECENT husband / partner. In what month and year was that?<br>*Select 'Do not know' for month and '2030' for year to indicate 'No Response'.*	
Month and Year	Month:<br>Year:

Check here if respondent does not know month.	☐

CHECK: Based on the response you entered in FFQ005a, the respondent was possibly 15 years old or younger at the beginning of her marriage or cohabitation. Did you enter FFQ005a correctly?	◯ Yes<br>◯ No
FFQ006. Does your husband / partner have other wives or does he live with other women as if married?	◯ Yes<br>◯ No<br>◯ Do not know<br>◯ No response
FFQ007. What is your religion?	◯ Protestant<br>◯ Orthodox<br>◯ Muslim<br>◯ Catholic<br>◯ Traditional<br>◯ Wakefeta<br>◯ Non-believers<br>◯ Other<br>◯ No response
FFQ008. What is the highest level of school you attended?<br>*Only record formal schooling. Do not record bible or koranic school or short courses.*	◯ Never attended<br>◯ Primary<br>◯ Secondary<br>◯ Technical & vocational<br>◯ Higher<br>◯ No response
FFQ009. Can you read or write in any language?	◯ Yes<br>◯ No<br>◯ No response

Section 2 – Migration

Now I would like to ask about your recent migration to and from your current area of residence

FFQ010. What region were you born in?	◯ Tigray<br>◯ Afar<br>◯ Amhara<br>◯ Oromia<br>◯ Ethiopia Somali<br>◯ Benishangul Gumuz<br>◯ SNNPR<br>◯ Gambella<br>◯ Harari<br>◯ Addis Ababa<br>◯ Dire Dawa<br>◯ Abroad

	◯ Do not know<br>◯ No response
FFQ011. Was the place where you were born rural or urban?	◯ Rural<br>◯ Urban<br>◯ Do not know<br>◯ No response
FFQ012. How long have you been living continuously in this district: ${district_name}?<br>*If recently moved (< 1 month to < 1 year), enter 'Months'. If a year or more, please enter in number of years on the following screen*	◯ Always<br>◯ Currently visiting<br>◯ Months<br>◯ Years<br>◯ No response
FFQ013. Enter number of ${duration_lab}<br>*If the respondent has stayed for less than a year 0 is a possible answer. Write '0' in the Month if they have recently moved (*	
FFQ014. Before you moved here, which region or country did you live in most recently?	◯ Tigray<br>◯ Afar<br>◯ Amhara<br>◯ Oromia<br>◯ Ethiopia Somali<br>◯ Benishangul Gumuz<br>◯ SNNPR<br>◯ Gambella<br>◯ Harari<br>◯ Addis Ababa<br>◯ Dire Dawa<br>◯ Yemen<br>◯ Saudi Arabia<br>◯ Libya<br>◯ Beirut<br>◯ United Arab Emirates<br>◯ Sudan<br>◯ South Sudan<br>◯ South Africa<br>◯ Kenya<br>◯ Lebanon<br>◯ Eritrea<br>◯ Other<br>◯ No response
FFQ015. Just before you moved here, did you live in a rural, or an urban area?	◯ Rural<br>◯ Urban<br>◯ Do not know<br>◯ No response
FFQ016. Who did you move with?<br>*Select all that apply*	☐ Partner<br>☐ Own parents<br>☐ Partner's parents<br>☐ Own children<br>☐ Other family members<br>☐ No one (moved alone)

	☐ Other<br>☐ No response
FFQ17. Can you tell me the main reason why you moved to your current place of residence?<br>*Select only one response*	◯ Education<br>◯ Search for work<br>◯ Marriage<br>◯ Divorce/Marriage dissolution<br>◯ Death of spouse<br>◯ Death of other household member<br>◯ Job transfer/have a job<br>◯ Displacement/War/drought<br>◯ Moved with family<br>◯ Returned back home<br>◯ Shortage of land<br>◯ Health problems<br>◯ Other<br>◯ No response

Section 3 – Reproduction & Fertility Preferences

Now I would like to ask about all the births you have had during your life.

FFQ018. Now I would like to ask about all the pregnancies you have had during your life. Have you ever been pregnant?	◯ Yes<br>◯ No<br>◯ No response
FFQ019. How many times have you given birth to a baby that was born alive?<br>*No response: -99*	

FFQ020. When was your FIRST birth?<br>*If respondent knows the year, but not month select the 'Does not know month' checkbox. Select the 'Do not know' checkbox for month and '2030' for year to indicate 'No Response'.*	
Month and Year	Month:<br>Year:
Check here if respondent does not know month.	☐

FFQ021. When was your MOST RECENT birth?<br>*Please record the date of the MOST RECENT live birth. The date should be found by calculating backwards from memorable events if needed. Select 'Do not know' for month and '2030' for year to indicate 'No Response'.*<br>*Use visual aid to record dates of most recent birth*	
Day, Month and Year	Day:<br>Month:

	Year:
Check here if respondent does not know month.	☐

FFQ023. Just before you moved here ${district_name}, did you have any sons or daughters whom you have given birth to?	◯ Yes<br>◯ No<br>◯ No response
FFQ024. How many children did you have just before you moved here?<br>*No response: -99*	
FFQ025. Are you pregnant now?	◯ Yes<br>◯ No<br>◯ Unsure<br>◯ No response
FFQ026. When did your last menstrual period start?<br>*Hint: Help the respondent to remember the approximate date by asking her usual menstrual cycle pattern and by using local languages which are equivalent to LMP*<br>*Enter 0 days for today*	◯ Days ago<br>◯ Weeks ago<br>◯ Months ago<br>◯ Years ago<br>◯ Before last birth<br>◯ Never menstruated<br>◯ In menopause/ has had hysterectomy<br>◯ No response
Enter in ${mens_period_lab}.	

FFQ029a. How many months pregnant are you?	
The most recent birth was: ${recent_birth_et_lab}	
Please record the number of completed months.<br>*No response: -99; Do not know: -88*	

FFQ029b. At the time you became pregnant, did you want to become pregnant then, did you want to wait until later, or did you not want to have any / any more children at all?<br>*Remind the respondent that we are asking about her most recent pregnancy, or if currently pregnant about the current pregnancy*	◯ Then<br>◯ Later<br>◯ Not at all<br>◯ No response
FFQ030. Have you ever delivered in a health facility before?	◯ Yes<br>◯ No<br>◯ No response
FFQ031. Based on your previous delivery experience, would you recommend that your friends or family members deliver in a facility or at home?	◯ Facility<br>◯ Home<br>◯ Do not know<br>◯ No response
FFQ032. Where would you like to deliver your baby?	◯ Her Home<br>◯ Other home

	◯ Government hospital<br>◯ Government health center<br>◯ Government health post<br>◯ Other public sector<br>◯ Private hospital/clinic<br>◯ Other private medical sector<br>◯ NGO/Faith-based health facility<br>◯ Other<br>◯ Have not decided yet<br>◯ No response
FFQ033. Who would you like to have help deliver your baby?	◯ Doctor<br>◯ Health officer<br>◯ Nurse/Midwife<br>◯ Any professional healthcare provider (can't distinguish)<br>◯ HEW<br>◯ Traditional birth attendant<br>◯ Family member<br>◯ No one<br>◯ Have not decided yet<br>◯ No response
FFQ034a. Thus far in your pregnancy, have you seen a health extension worker for antenatal care?	◯ Yes<br>◯ No<br>◯ Do not know<br>◯ No response
FFQ034b. At any point in your pregnancy, did you see a health extension worker for antenatal care?	◯ Yes<br>◯ No<br>◯ Do not know<br>◯ No response
FFQ035. Where did you see the HEW?	☐ Home<br>☐ Government health post<br>☐ Other health facility<br>☐ Other<br>☐ No response
FFQ036a. How many times have you received antenatal care during this pregnancy from a health extension worker, either at a health post or at home?<br>*No response: -99; Do not know: -88*	
FFQ036b. How many times did you receive antenatal care during this pregnancy from a health extension worker, either at a health post or at home?<br>*No response: -99; Do not know: -88*	
FFQ037. How many months pregnant were you when you first talked to a health extension worker about your pregnancy?<br>*No response: -99; Do not know: -88*	

FFQ038a. Have you seen a professional healthcare provider, other than an HEW, for antenatal care during this pregnancy?	◯ Yes<br>◯ No<br>◯ No response
FFQ038b. Did you see a professional healthcare provider, other than an HEW, for antenatal care during this pregnancy?	◯ Yes<br>◯ No<br>◯ No response
FFQ039. Whom did you see, not including an HEW? Anyone else?<br>*Select all that apply Probe to identify each type of person and record all mentioned.*	☐ Doctor<br>☐ Health officer<br>☐ Nurse/Midwife<br>☐ Professional health provider, can't distinguish<br>☐ Other<br>☐ No response
FFQ040a. How many times have you received antenatal care during this pregnancy from a professional healthcare provider, other than an HEW?<br>*No response: -99; Do not know: -88*	
FFQ040b. How many times did you receive antenatal care during this pregnancy from a professional healthcare provider, other than an HEW?<br>*No response: -99; Do not know: -88*	
FFQ041. How many months pregnant were you when you first received antenatal from a professional healthcare provider other than an HEW for this pregnancy?<br>*No response: -99; Do not know: -88*	
FFQ042. Where did you receive antenatal care for this pregnancy, not including from the HEW? Anywhere else?<br>*Select all that apply*<br>*Probe to identify the type of source and record all mentioned*	☐ Her Home<br>☐ Other home<br>☐ Government hospital<br>☐ Government health center<br>☐ Government health post<br>☐ Other public sector<br>☐ Private hospital/clinic<br>☐ Other private medical sector<br>☐ NGO/Faith-based health facility<br>☐ Traditional healer/medicine<br>☐ Other<br>☐ Nowhere, no treatment sought<br>☐ No response

FFQ043. As part of your antenatal care during this pregnancy were any of the following measured at least once:<br>*Hint: This includes any ANC from any provider*	
A) Was your blood pressure measured?	◯ Yes<br>◯ No

	◯ Do not know<br>◯ No response
B) Was your weight taken?	◯ Yes<br>◯ No<br>◯ Do not know<br>◯ No response
C) Did you give a urine sample that was not for a pregnancy test?	◯ Yes<br>◯ No<br>◯ Do not know<br>◯ No response
D) Did you give a blood sample?	◯ Yes<br>◯ No<br>◯ Do not know<br>◯ No response
E) Did you give a stool sample?	◯ Yes<br>◯ No<br>◯ Do not know<br>◯ No response

FFQ044. I don't want to know the results, but as part of your antenatal care were you:<br>*This includes any ANC from any provider*	
A) Tested for syphilis?	◯ Yes<br>◯ No<br>◯ Do not know<br>◯ No response
B) Did you receive the results of your test?	◯ Yes<br>◯ No<br>◯ Do not know<br>◯ No response
C) Did someone discuss the results with you after you were tested?	◯ Yes<br>◯ No<br>◯ Do not know<br>◯ No response
FFQ045. I don't want to know the results, but as part of your antenatal care were you:<br>*This includes any ANC from any provider*	
A) Tested for HIV?	◯ Yes<br>◯ No<br>◯ Do not know<br>◯ No response
B) Did you receive the results of your test?	◯ Yes<br>◯ No<br>◯ Do not know<br>◯ No response

C) Did someone discuss the results with you after you were tested?	◯ Yes<br>◯ No<br>◯ Do not know<br>◯ No response
FFQ046a. During your antenatal care visit, did your provider discuss breastfeeding as a method to prevent pregnancy?<br>*This includes any ANC from any provider*	◯ Yes<br>◯ No<br>◯ Do not know<br>◯ No response
FFQ046b. During your antenatal care visit, did your provider talk with you about postpartum family planning?<br>*This includes any ANC from any provider.*	◯ Yes<br>◯ No<br>◯ Do not know<br>◯ No response
FFQ047. Which family planning method or methods did you discuss with the provider?<br>*Select all that apply*<br>*Note: breastfeeding was included previously and is not part of this list*	☐ Female Sterilization<br>☐ Male Sterilization<br>☐ Implant<br>☐ IUD<br>☐ Injectables<br>☐ Pill<br>☐ Emergency Contraception<br>☐ Male Condom<br>☐ Female Condom<br>☐ Std. Days/Cycle beads<br>☐ Rhythm method<br>☐ Withdrawal<br>☐ Other traditional methods<br>☐ No response
FFQ048. Are you planning to breastfeed?	◯ Yes<br>◯ No<br>◯ Not sure yet<br>◯ No response
FFQ049. Are you planning to use breastfeeding to delay or avoid getting pregnant?	◯ Yes<br>◯ No<br>◯ Not sure yet<br>◯ No response
FFQ050. How long do you plan to breastfeed?	◯ Months<br>◯ Years<br>◯ Not sure yet<br>◯ No response
Enter in ${brfeeding_period_lab}.	
FFQ051. Do you plan to feed your baby anything other than breastmilk in the first six months? This includes things like water, juice, oil, or tea.	◯ Yes<br>◯ No<br>◯ Not sure yet<br>◯ No response
FFQ052. Do you plan to use a method of family planning, other than breastfeeding, within a year of giving birth?	◯ Yes<br>◯ No

	◯ Do not know<br>◯ No response
FFQ053a. When do you plan to start using the method?	◯ Immediately (at facility)<br>◯ At first postpartum visit<br>◯ Weeks<br>◯ Months<br>◯ Not sure yet<br>◯ No response
FFQ053b. Enter value in ${start_method_lab}	
FFQ054. What method do you plan to use?<br>*Hint: this does not include LAM/breastfeeding*	◯ Female Sterilization<br>◯ Male Sterilization<br>◯ Implant<br>◯ IUD<br>◯ Injectables<br>◯ Pill<br>◯ Emergency Contraception<br>◯ Male Condom<br>◯ Female Condom<br>◯ Std. Days/Cycle beads<br>◯ Rhythm method<br>◯ Withdrawal<br>◯ Other traditional methods<br>◯ Not sure yet<br>◯ No response
FFQ055. During this pregnancy, were you given an injection in the arm to prevent the baby from getting tetanus, that is convulsions (locked jaw) after birth?	◯ Yes<br>◯ No<br>◯ Do not know<br>◯ No response
FFQ056. During this pregnancy, how many times have you gotten a tetanus injection?	
CHECK: You entered that the respondent received ${tetanus_inj_times} tetanus injections in question FFQ056. Confirm that these were received only during this pregnancy.	
FFQ057. During this pregnancy have you consumed any multivitamins that contain iron, iron tablets, iron syrup, or folic acid/iron co-packs?<br>*A photo of iron tablets/syrup will appear on the screen*<br>[iron_tablets_syrup.png]	◯ Yes<br>◯ No<br>◯ Do not know<br>◯ No response
FFQ058. During this pregnancy, have you consumed any drug for intestinal worms?<br>*A photo of intestinal worms tablets will appear on the screen*<br>[albendazole.png]	◯ Yes<br>◯ No<br>◯ Do not know<br>◯ No response

FFQ059a. Have you experienced any of the following problems during this pregnancy:	

A) Severe headache with blurred vision?	◯ Yes<br>◯ No<br>◯ Do not know<br>◯ No response
B) High blood pressure?	◯ Yes<br>◯ No<br>◯ Do not know<br>◯ No response
C) Edema (swelling) face/feet/body?	◯ Yes<br>◯ No<br>◯ Do not know<br>◯ No response
D) Convulsion/fits?	◯ Yes<br>◯ No<br>◯ Do not know<br>◯ No response
E) Vaginal bleeding before delivery?	◯ Yes<br>◯ No<br>◯ Do not know<br>◯ No response
F) High fever?	◯ Yes<br>◯ No<br>◯ Do not know<br>◯ No response
G) Abnormal vaginal discharge (foul smelling/dark)?	◯ Yes<br>◯ No<br>◯ Do not know<br>◯ No response
H) Lower abdominal pain?	◯ Yes<br>◯ No<br>◯ Do not know<br>◯ No response
I) Worsening vision, particularly at night?	◯ Yes<br>◯ No<br>◯ Do not know<br>◯ No response

FFQ059b. Did you experience any of the following problems during this pregnancy:	
A) Severe headache with blurred vision?	◯ Yes<br>◯ No<br>◯ Do not know<br>◯ No response

B) High blood pressure?	◯ Yes<br>◯ No<br>◯ Do not know<br>◯ No response
C) Edema (swelling) face/feet/body?	◯ Yes<br>◯ No<br>◯ Do not know<br>◯ No response
D) Convulsion/fits?	◯ Yes<br>◯ No<br>◯ Do not know<br>◯ No response
E) Vaginal bleeding before delivery?	◯ Yes<br>◯ No<br>◯ Do not know<br>◯ No response
F) High fever?	◯ Yes<br>◯ No<br>◯ Do not know<br>◯ No response
G) Abnormal vaginal discharge (foul smelling/dark)?	◯ Yes<br>◯ No<br>◯ Do not know<br>◯ No response
H) Lower abdominal pain?	◯ Yes<br>◯ No<br>◯ Do not know<br>◯ No response
I) Worsening vision, particularly at night?	◯ Yes<br>◯ No<br>◯ Do not know<br>◯ No response

FFQ060A. Did you seek treatment at a health facility for Severe headache with blurred vision?	◯ Yes<br>◯ No<br>◯ No response
FFQ060B. Did you seek treatment at a health facility for High blood pressure?	◯ Yes<br>◯ No<br>◯ No response
FFQ060C. Did you seek treatment at a health facility for Edema face/feet/body?	◯ Yes<br>◯ No<br>◯ No response

FFQ060D. Did you seek treatment at a health facility for Convulsion/fits?	◯ Yes<br>◯ No<br>◯ No response
FFQ060E. Did you seek treatment at a health facility for Vaginal bleeding before delivery?	◯ Yes<br>◯ No<br>◯ No response
FFQ060F. Did you seek treatment at a health facility for High fever?	◯ Yes<br>◯ No<br>◯ No response
FFQ060G. Did you seek treatment at a health facility for Abnormal vaginal discharge (foul smelling/dark)?	◯ Yes<br>◯ No<br>◯ No response
FFQ060H. Did you seek treatment at a health facility for Lower abdominal pain?	◯ Yes<br>◯ No<br>◯ No response
FFQ060I. Did you seek treatment at a health facility for Difficulty seeing at night?	◯ Yes<br>◯ No<br>◯ No response

FFQ061. During your antenatal care visit(s) was there any discussion about the following:	
A) Place of delivery?	◯ Yes<br>◯ No<br>◯ Do not know<br>◯ No response
B) Delivery by a skilled attendant?	◯ Yes<br>◯ No<br>◯ Do not know<br>◯ No response
C) Arrangement for transport for delivery?	◯ Yes<br>◯ No<br>◯ Do not know<br>◯ No response
D) Where to go if experience of pregnancy danger signs?	◯ Yes<br>◯ No<br>◯ Do not know<br>◯ No response
E) Severe headaches with blurred vision as a danger sign in pregnancy?	◯ Yes<br>◯ No<br>◯ Do not know<br>◯ No response
F) High blood pressure as a danger sign in pregnancy?	◯ Yes<br>◯ No

	◯ Do not know<br>◯ No response
G) Edema/swelling of the face/feet/body as a danger sign in pregnancy?	◯ Yes<br>◯ No<br>◯ Do not know<br>◯ No response
H) Convulsions/fits as a danger sign in pregnancy?	◯ Yes<br>◯ No<br>◯ Do not know<br>◯ No response
I) Bleeding before delivery as a danger sign in pregnancy?	◯ Yes<br>◯ No<br>◯ Do not know<br>◯ No response

FFQ062. Did you receive any tablets that should be taken to prevent bleeding after delivery?	◯ Yes<br>◯ No<br>◯ Do not know<br>◯ No response
FFQ063. As part of your antenatal care visits, did a healthcare worker or HEW talk with you about your nutrition or diet?	◯ Yes<br>◯ No<br>◯ Do not know<br>◯ No response
FFQ064. What information or messages did you receive during your pregnancy about nutrition or diet?<br>*Probe: From either a health provider at a facility or an HEW?*<br>*Select all that apply. Read all responses aloud.*	☐ Eat more (quantity)<br>☐ Eat a variety of foods / foods rich with iron (quality)<br>☐ Take iron-containing tablets (IFAS)<br>☐ Take preventive malaria treatment<br>☐ Take deworming tablet<br>☐ How much weight to gain<br>☐ Regularly exercise<br>☐ How to manage nausea/vomiting<br>☐ Reduce salt intake<br>☐ Do not eat raw meat<br>☐ None of the above<br>☐ No response
FFQ065. During this pregnancy, did you participate in a 1 to 5 meeting to discuss pregnancy-related issues with your team or team leader?	◯ Yes<br>◯ No, member but did not participate<br>◯ No, not member<br>◯ No response
FFQ066. Do you know how to contact the HEW if you go into labor?	◯ Yes<br>◯ No<br>◯ No HEW<br>◯ No response

FFQ067a. Has your partner encouraged you to go to the clinic for antenatal care?	◯ Yes, encouraged<br>◯ No, did not encourage<br>◯ No, actively discouraged<br>◯ No partner<br>◯ Do not know<br>◯ No response
FFQ067b. Did your partner encourage you to go to the clinic for antenatal care?	◯ Yes<br>◯ No, did not encourage<br>◯ No, actively discouraged<br>◯ No partner<br>◯ Do not know<br>◯ No response
FFQ068. Have you and your partner discussed where you are planning to deliver?	◯ Yes<br>◯ No<br>◯ Do not know<br>◯ Partner not involved<br>◯ No response
FFQ069. While you were pregnant, did you and your partner discuss where you planned to deliver?	◯ Yes<br>◯ No<br>◯ Do not know<br>◯ Partner not involved<br>◯ No response
FFQ148a. You said you are currently pregnant<br>How many other pregnancies have you had since September 2017?<br>*For each pregnancy in the last two years, add a group Use New Year as a reference, Max # pregnancies are 4*	
FFQ148b. How many pregnancies have you had since September 2017?<br>*If the respondent is recently postpartum, i.e. had a birth in the last 8 weeks please record all other pregnancies that she has had in the last two years. Do not include the pregnancy that she has already told you about in FFQ071*	
FFQ151. At the time you became pregnant, did you want to become pregnant then, did you want to wait until later, or did you not want to have any / any more children at all?	◯ Then<br>◯ Later<br>◯ Not at all<br>◯ No response
Now think back to your most recent pregnancy (current pregnancy if currently pregnant).	
FFQ169. In terms of becoming a mother (first time or again), you feel that your pregnancy happened at the......<br>*Read the response options.*	◯ Right time<br>◯ Ok, but not quite right time<br>◯ Wrong time<br>◯ No response
FFQ170a. Just before you became pregnant.......<br>*Read the response options.*	◯ You intended to get pregnant<br>◯ Your intentions kept changing

	◯ You did not intend to get pregnant<br>◯ No response
FFQ170b. Just before you became pregnant....<br>*Read the response options.*	◯ You wanted to have a baby<br>◯ You had mixed feelings about having a baby<br>◯ You did not want to have a baby<br>◯ No response
FFQ171. Before you became pregnant....<br>*Read the response options.*	◯ You and your partner had agreed for you to get pregnant<br>◯ You and your partner had discussed having children together, but hadn't agreed for you to get pregnant<br>◯ You and your partner never discussed having children together<br>◯ No response
FFQ172. When you found out you were pregnant, how did you feel?<br>*Read the response options*	◯ Very happy<br>◯ Sort of happy<br>◯ Mixed happy and unhappy<br>◯ Sort of unhappy<br>◯ Very unhappy<br>◯ No response
FFQ173. When your partner found out you were pregnant, how did he feel?<br>*Read the response options*	◯ Very happy<br>◯ Sort of happy<br>◯ Mixed happy and unhappy<br>◯ Sort of unhappy<br>◯ Very unhappy<br>◯ Have not told partner<br>◯ No partner<br>◯ Do not know<br>◯ No response
FFQ174. Before you became pregnant, did you do any of the following in preparation for pregnancy?<br>*Read the response options Select multiple*	☐ Took folic acid/vitamins<br>☐ Ate more healthily<br>☐ Sought medical/health advice<br>☐ Saved money for healthcare<br>☐ You did not do any of the above before your pregnancy<br>☐ No response
Now I have some questions about the future	
FFQ175a. Would you like to have a/another child or would you prefer not to have any / any more children?	◯ Have a/another child<br>◯ No more/ prefer no children<br>◯ Says she can't get pregnant<br>◯ Undecided/Don't know<br>◯ No response

FFQ175b. After the child you are expecting now, would you like to have another child, or would you prefer not to have any more children?	◯ Have a/another child<br>◯ No more/ prefer no children<br>◯ Undecided/Don't know<br>◯ No response
FFQ176a. How long would you like to wait from now before the birth of a/another child?<br>*If you select months or years, you will enter a number for x on the next screen. Select "Years" if more than 36 months. Please check that you correctly entered the value for months/years.*	◯ Months<br>◯ Years<br>◯ Soon / Now<br>◯ Other<br>◯ Do not know<br>◯ No response
Enter in ${birth_gap_lab}	
FFQ176b. After the birth of the child you are expecting now, how long would you like to wait before the birth of another child?<br>*If you select months or years, you will enter a number for x on the next screen. Select "Years" if more than 36 months. Please check that you correctly entered the value for months/years.*	◯ Months<br>◯ Years<br>◯ Soon / Now<br>◯ Other<br>◯ Do not know<br>◯ No response
Enter in ${current_birth_gap_lab}	
FFQ177. If you got pregnant now, how would you feel?	◯ Very happy<br>◯ Sort of happy<br>◯ Mixed happy and unhappy<br>◯ Sort of unhappy<br>◯ Very unhappy<br>◯ No response

Section 4 – Contraception

Now I would like to talk about family planning - the various ways or methods that a couple can use to delay or avoid a pregnancy.

An image will appear on the screen for some methods. If the respondent says that she has not heard of the method or if she hesitates to answer, read the probe aloud and show her the image, if available.

FFQ178a. Have you ever heard of female sterilization?<br>*PROBE: Women can have an operation to avoid having any more children.*	◯ Yes<br>◯ No<br>◯ No response
FFQ178b. Have you ever heard of male sterilization?<br>*PROBE: Men can have an operation to avoid having any more children.*	◯ Yes<br>◯ No<br>◯ No response
FFQ178c. Have you ever heard of the contraceptive implant?<br>*PROBE: Women can have one or several small rods placed in their upper arm by a doctor or nurse, which can prevent pregnancy for one or more years*<br>[implant.png]	◯ Yes<br>◯ No<br>◯ No response

FFQ178d. Have you ever heard of the IUD?<br>*PROBE: Women can have a loop or coil placed inside them by a doctor or a nurse.*<br>[IUD_150x300.png]	◯ Yes<br>◯ No<br>◯ No response
FFQ178e. Have you ever heard of injectables?<br>*PROBE: Women can have an injection by a health provider that stops them from becoming pregnant for one or more months.*<br>[injectable_150x300.png]	◯ Yes<br>◯ No<br>◯ No response
FFQ178f. Have you ever heard of the (birth control) pill?<br>*PROBE: Women can take a pill every day to avoid becoming pregnant.*<br>[pill_150x300.png]	◯ Yes<br>◯ No<br>◯ No response
FFQ178g. Have you ever heard of emergency contraception?<br>*PROBE: As an emergency measure after unprotected sexual intercourse women can take special pills at any time within five days to prevent pregnancy.*	◯ Yes<br>◯ No<br>◯ No response
FFQ178h. Have you ever heard of condoms?<br>*PROBE: Men can put a rubber sheath on their penis before sexual intercourse.*<br>[male_condom_150x300.png]	◯ Yes<br>◯ No<br>◯ No response
FFQ178i. Have you ever heard of female condoms?<br>*PROBE: Women can put a sheath in their vagina before sexual intercourse.*<br>[female_condom_150x300.png]	◯ Yes<br>◯ No<br>◯ No response
FFQ178j. Have you ever heard of the standard days method or Cycle Beads?<br>*PROBE: A Woman can use a string of colored beads to know the days she can get pregnant. On the days she can get pregnant, she and her partner use a condom or do not have sexual intercourse.*<br>[SDM-Beads_only.png]	◯ Yes<br>◯ No<br>◯ No response
FFQ178k. Have you ever heard of the Lactational Amenorrhea Method or LAM?	◯ Yes<br>◯ No<br>◯ No response
FFQ178L. Have you ever heard of the rhythm method?<br>*PROBE: Women can avoid pregnancy by not having sexual intercourse on the days of the month they think they can get pregnant.*	◯ Yes<br>◯ No<br>◯ No response
FFQ178M. Have you ever heard of the withdrawal method?<br>*PROBE: Men can be careful and pull out before climax.*	◯ Yes<br>◯ No<br>◯ No response
FFQ178n. Have you ever heard of any other ways or methods that women or men can use to avoid pregnancy?	◯ Yes<br>◯ No<br>◯ No response
FFQ179a. Are you or your partner currently doing something or using any method to delay or avoid getting pregnant?	◯ Yes<br>◯ No<br>◯ No response

FFQ179b. Which method or methods are you using?<br>*Probe: Anything else? Select all methods mentioned. Be sure to scroll to bottom to see all choices.*	☐ Female Sterilization<br>☐ Male Sterilization<br>☐ Implant<br>☐ IUD<br>☐ Injectables<br>☐ Pill<br>☐ Emergency Contraception<br>☐ Male Condom<br>☐ Female Condom<br>☐ Std. Days/Cycle beads<br>☐ LAM<br>☐ Rhythm method<br>☐ Withdrawal<br>☐ Other traditional methods<br>☐ No response

FFQ180. Since what months and years have you been using ${current_recent_method_lab} without stopping?<br>*Calculate backwards from memorable events if needed.*	
#####<br>*Most Recent Birth: ${recent_birth_et_lab}.*	
#####<br>*Current Marriage: ${current_marriage_date_et_lab}.*	
#####<br>*Must be before today. Respondent must be at least 10 years old. Select 'Do not know' for month and '2030' for year to indicate 'No Response'.*<br>*For RE: Mark start date and all months until now as current use on the visual aid*	
Month and Year	Month:<br>Year:
Check here if respondent does not know month.	☐

FFQ181. Did you or your partner use any other methods between September 2017 and when you started using ${current_method_lab_cs}?<br>*Interviewer notes: [Since September 2017; use New Year as a reference]. Probe to see if she has used the same method at a previous time.*	◯ Yes<br>◯ No<br>◯ No response
CS1. Have you or your partner done anything or used a method to delay or avoid getting pregnant in the last 2 years (since September 2017)?<br>*Interviewer note: [Since September 2017; use New Year as a reference]*	◯ Yes<br>◯ No<br>◯ No response

Please swipe forward and select "ADD GROUP" to Start and Stop Dates for this method.	

Contraceptive Methods

ERROR: Too many groups added.<br>*Remove this group by pressing your finger down on the small circle below and selecting 'remove group' from the menu that appears.*	
#####	◯

CS2. Which method did you use just before ${current_method_lab_cs}?<br>*Do not include the current method of use. Only select the current method if the woman used it continuously, stopped and restarted during the two year period.*	
CS2. Which method did you use?<br>*Do not include the current method of use. Only select the current method if the woman used it continuously, stopped and restarted during the two year period.*	
#####	◯ Female Sterilization<br>◯ Male Sterilization<br>◯ Implant<br>◯ IUD<br>◯ Injectables<br>◯ Pill<br>◯ Emergency Contraception<br>◯ Male Condom<br>◯ Female Condom<br>◯ Std. Days/Cycle beads<br>◯ LAM<br>◯ Rhythm method<br>◯ Withdrawal<br>◯ Other traditional methods<br>◯ No response

FFQ182. When did you stop using your ${cs2_method_lab}?<br>*If respondent knows the year, but not month select the 'Does not know month' checkbox. Select the 'Do not know' checkbox for month and '2030' for year to indicate 'No Response'*	
Month and Year	Month:<br>Year:
Check here if respondent does not know month.	☐

FFQ183. When did you start using ${cs2_method_lab}?<br>*Please indicate the year and month you started using it.*<br>*Calculate backwards from memorable events if needed.*	
#####<br>*Most Recent Birth: ${recent_birth_et_lab}.*	
#####<br>*Current Marriage: ${current_marriage_date_et_lab}.*	
#####<br>*If respondent knows the year, but not month select the 'Does not know month' checkbox. Select the 'Do not know' checkbox for month and '2030' for year to indicate 'No Response'.*	
Month and Year	Month:<br>Year:
Check here if respondent does not know month.	☐
FFQ184. You said you did not know when you started using ${cs2_method_lab}.<br>Can you remember about how long you used ${cs2_method_lab} for?<br>*Number of months:*	
FFQ185. Before ${cs2_method_lab}, did you or your partner use anything else to delay or avoid getting pregnant since September 2017?<br>*ODK will keep repeating these questions until the start and stop dates for a given method are both before September 2017 OR until response is "NONE"*	◯ Yes<br>◯ No<br>◯ Do not know<br>◯ No response
There are other method used. Move forward and select "Add Group"	
There are no other method used. Move forward and select "Do Not Add"	
If there are any more methods to add, move forward and select "Add Group". Otherwise, select "Do not Add.	
Warning: The respondent indicated that she or her partner did something or used a method to delay or avoid getting pregnant in the last 2 years (since September 2017). But you did not add a "Contraceptive Methods" group. Please go back to CS1 and check your response.	
SUMMARY	

Please review with the respondent that all information is correct.	
Current pregnancy start: ${current_preg_start_et_lab}	
Most recent birth: ${recent_birth_start_et_lab} - ${recent_birth_et_lab}	
First birth: ${first_birth_start_et_lab} - ${first_birth_et_lab}	
No previous births	
Current method: ${current_method_lab_cs}. Start date: ${start_mtd_date_et_lab}.	
${full_summary_et}	
Method 1: ${cs_summary_mtd_name1}. Start Date: ${cs_summary_mtd_start1}. Stop Date: ${cs_summary_mtd_stop1}.	
Method 2: ${cs_summary_mtd_name2}. Start Date: ${cs_summary_mtd_start2}. Stop Date: ${cs_summary_mtd_stop2}.	
Method 3: ${cs_summary_mtd_name3}. Start Date: ${cs_summary_mtd_start3}. Stop Date: ${cs_summary_mtd_stop3}.	
Method 4: ${cs_summary_mtd_name4}. Start Date: ${cs_summary_mtd_start4}. Stop Date: ${cs_summary_mtd_stop4}.	
Method 5: ${cs_summary_mtd_name5}. Start Date: ${cs_summary_mtd_start5}. Stop Date: ${cs_summary_mtd_stop5}.	
No current or past method use	

CURRENT/RECENT USERS: I will ask you a few more questions on the method that you are currently using or the method you used most recently.	
FFQ186. Did the provider tell you or your partner that this method was permanent?	◯ Yes<br>◯ No<br>◯ No response
FFQ187. You first started using ${current_recent_method_lab} in ${start_mtd_date_et_lab}. Where did you or your partner get it at that time?<br>*Scroll to bottom to see all choices.*	◯ GOVT HOSPITAL<br>◯ GOVT HEALTH CENTER<br>◯ GOVT. HEALTH POST/HEW<br>◯ Health development army<br>◯ Public Pharmacy<br>◯ OTHER PUBLIC<br>◯ NGO HEALTH FACILITY<br>◯ OTHER NGO<br>◯ PRIVATE HOSPITAL<br>◯ PRIVATE CLINIC<br>◯ Private PHARMACY

	◯ OTHER PRIVATE MEDICAL<br>◯ DRUG VENDORS/STORE<br>◯ Shop<br>◯ FRIEND/RELATIVE<br>◯ Self<br>◯ OTHER<br>◯ DO not KNOW<br>◯ NO RESPONSE
FFQ188. When you obtained your ${current_recent_method_lab}, did the provider ask you about your prior experience with contraception?	◯ Yes<br>◯ No<br>◯ Do not know<br>◯ No response
FFQ189. When you obtained your ${current_recent_method_lab}, did you obtain the method you wanted to use to delay or avoid getting pregnant?	◯ Yes<br>◯ No<br>◯ No response
FFQ190. Why did you choose the implant?<br>*Select multiple*	☐ Long duration of protection<br>☐ Less need for follow-up<br>☐ Unavailability of other methods<br>☐ Provider recommended<br>☐ Other<br>☐ No response
FFQ191. At the visit when the implant was inserted, were you told for how long the implant would protect you from pregnancy?	◯ Yes<br>◯ No<br>◯ No response
FFQ192. Were you told where you could go to have the implant removed?	◯ Yes<br>◯ No<br>◯ No response
FFQ193. Were you told how much it would cost to get your implant removed?	◯ Yes<br>◯ No<br>◯ No response
FFQ194. When you obtained your ${current_recent_method_lab}, were you told by the provider about side effects or problems you might have with a method to delay or avoid pregnancy?	◯ Yes<br>◯ No<br>◯ No response
FFQ195. According to the provider what are the possible side effects or problems related to use of ${current_recent_method_lab}?	☐ Less bleeding or no bleeding<br>☐ Heavier bleeding<br>☐ Irregular bleeding<br>☐ spotting/bleeding<br>☐ Non-specific bleeding changes<br>☐ Uterine cramping/lower abdominal pain<br>☐ Gained weight<br>☐ Lost weight<br>☐ Facial spotting/facial pigmentation<br>☐ Headaches<br>☐ Got infection

	☐ Nausea/vomiting<br>☐ Increased menstrual cramping<br>☐ Lowered sex drive<br>☐ Vaginal dryness<br>☐ Infertility/sterility<br>☐ Delayed return to fertility<br>☐ Method get lost inside body<br>☐ General weakness<br>☐ Diarrhea<br>☐ OTHER<br>☐ DO not KNOW<br>☐ NO RESPONSE
FFQ196. Were you told what to do if you experienced these side effects or problems?	◯ Yes<br>◯ No<br>◯ No response
FFQ197. At that time, were you told by a family planning provider about methods of family planning other than ${current_recent_method_lab} that you could use?	◯ Yes<br>◯ No<br>◯ No response
FFQ198. What methods were you told about?<br>*Select multiple*	☐ Female Sterilization<br>☐ Male Sterilization<br>☐ Implant<br>☐ IUD<br>☐ Injectables<br>☐ Pill<br>☐ Emergency Contraception<br>☐ Male Condom<br>☐ Female Condom<br>☐ Std. Days/Cycle beads<br>☐ LAM<br>☐ Rhythm method<br>☐ Withdrawal<br>☐ No response
FFQ199. At that time, were you told that you could switch to a different method in the future?	◯ Yes<br>◯ No<br>◯ No response
FFQ200. During that visit, who made the final decision about what method you got?	◯ You alone<br>◯ Provider<br>◯ Partner<br>◯ You and provider<br>◯ You and partner<br>◯ Other<br>◯ Do not know<br>◯ No response
FFQ201. Would you return to this provider? Provider: ${recent_mth_source_en}	◯ Yes<br>◯ No<br>◯ Do not know<br>◯ No response

FFQ202. Would you refer your relative or friend to this provider / facility?<br>Provider: ${recent_mth_source_en}	◯ Yes<br>◯ No<br>◯ Do not know<br>◯ No response
FFQ203. Are you/did you experience any of the following changes in your menstrual cycle due to ${current_recent_method_lab}?<br>*Read all options out loud. Select all that apply.*	☐ Less bleeding or no bleeding<br>☐ Heavier bleeding<br>☐ Irregular bleeding/spotting<br>☐ No change<br>☐ OTHER<br>☐ Do not know<br>☐ No response
FFQ204. How worried are/were you about these changes?	◯ Very worried<br>◯ A little worried<br>◯ Not at all worried<br>◯ No response
FFQ205. Are you experiencing any side effects?	◯ Yes<br>◯ No<br>◯ No response
FFQ205. Did you experience any side effects?	◯ Yes<br>◯ No<br>◯ No response
FFQ206. What are the side effects you are currently experiencing?<br>*Do not read option choices aloud*	☐ Less bleeding or no bleeding<br>☐ Heavier bleeding<br>☐ Irregular bleeding/spotting<br>☐ Uterine cramping/lower abdominal pain<br>☐ Gained weight<br>☐ Lost weight<br>☐ Facial spotting<br>☐ Headaches<br>☐ Got infection<br>☐ Nausea/vomiting<br>☐ Increased menstrual cramping<br>☐ Lowered sex drive<br>☐ Decreased sexual pleasure<br>☐ Vaginal dryness<br>☐ General weakness/pain<br>☐ Diarrhea<br>☐ Partner feels during sex<br>☐ Pain at insertion site<br>☐ Mood swings<br>☐ Backache<br>☐ OTHER<br>☐ DO not KNOW<br>☐ NO RESPONSE
FFQ206. What were the side effects that you EXPERIENCED while using the method?	☐ Less bleeding or no bleeding<br>☐ Heavier bleeding

Do not read option choices aloud	☐ Irregular bleeding/spotting<br>☐ Uterine cramping/lower abdominal pain<br>☐ Gained weight<br>☐ Lost weight<br>☐ Facial spotting<br>☐ Headaches<br>☐ Got infection<br>☐ Nausea/vomiting<br>☐ Increased menstrual cramping<br>☐ Lowered sex drive<br>☐ Decreased sexual pleasure<br>☐ Vaginal dryness<br>☐ General weakness/pain<br>☐ Diarrhea<br>☐ Partner feels during sex<br>☐ Pain at insertion site<br>☐ Mood swings<br>☐ Backache<br>☐ OTHER<br>☐ DO not KNOW<br>☐ NO RESPONSE
FFQ207. Are there any side effects that you are WORRIED ABOUT EXPERIENCING while using this method, but are not actually experiencing?	◯ Yes<br>◯ No<br>◯ No response
FFQ207. Were there any side effects that you WERE WORRIED ABOUT EXPERIENCING while using this method, but did not actually experience?	◯ Yes<br>◯ No<br>◯ No response
FFQ208. What are the side effects that you ARE WORRIED ABOUT EXPERIENCING while using this method, but are not actually experiencing?<br>*Do not read option choices aloud*	☐ Less bleeding or no bleeding<br>☐ Heavier bleeding<br>☐ Irregular bleeding/spotting<br>☐ Uterine cramping/lower abdominal pain<br>☐ Weight change<br>☐ Facial spotting<br>☐ Headaches<br>☐ General weakness/pain<br>☐ Pain at insertion site<br>☐ Got infection<br>☐ Method gets lost inside body<br>☐ Nausea/vomiting<br>☐ Lowered sex drive<br>☐ Decreased sexual pleasure<br>☐ Vaginal dryness<br>☐ Partner feels during sex<br>☐ Infertility/sterility<br>☐ Delayed return to fertility<br>☐ Deformation of babies<br>☐ Diarrhea

	☐ Cancer/fibroids<br>☐ Blood build up/impurities<br>☐ Pills accumulate in body<br>☐ Increased hair growth<br>☐ Mood swings<br>☐ OTHER<br>☐ DO not KNOW<br>☐ NO RESPONSE
FFQ208. What were the side effects that you WERE WORRIED ABOUT EXPERIENCING while using this method, but did not actually experience?<br>*Do not read option choices aloud*	☐ Less bleeding or no bleeding<br>☐ Heavier bleeding<br>☐ Irregular bleeding/spotting<br>☐ Uterine cramping/lower abdominal pain<br>☐ Weight change<br>☐ Facial spotting<br>☐ Headaches<br>☐ General weakness/pain<br>☐ Pain at insertion site<br>☐ Got infection<br>☐ Method gets lost inside body<br>☐ Nausea/vomiting<br>☐ Lowered sex drive<br>☐ Decreased sexual pleasure<br>☐ Vaginal dryness<br>☐ Partner feels during sex<br>☐ Infertility/sterility<br>☐ Delayed return to fertility<br>☐ Deformation of babies<br>☐ Diarrhea<br>☐ Cancer/fibroids<br>☐ Blood build up/impurities<br>☐ Pills accumulate in body<br>☐ Increased hair growth<br>☐ Mood swings<br>☐ OTHER<br>☐ DO not KNOW<br>☐ NO RESPONSE

FFQ209. The last time you received your ${current_recent_method_lab}, did you have to pay out of pocket for:	
A. Medical Card?	◯ Yes<br>◯ No<br>◯ Do not know<br>◯ No response
B. Supplies (like gloves or syringes)	◯ Yes<br>◯ No<br>◯ Do not know<br>◯ No response

C. The method itself?	◯ Yes<br>◯ No<br>◯ Do not know<br>◯ No response
D. Transportation?	◯ Yes<br>◯ No<br>◯ Do not know<br>◯ No response

FFQ210. Do you want to have your implant removed?	◯ Yes<br>◯ No<br>◯ No response
FFQ211a. In the past 12 months, did you try to have your current implant removed?	◯ Yes<br>◯ No<br>◯ No response
FFQ211b. Where did you go to try to have your implant removed?	◯ GOVT HOSPITAL<br>◯ GOVT HEALTH CENTER<br>◯ GOVT. HEALTH POST/HEW<br>◯ Health development army<br>◯ Public Pharmacy<br>◯ OTHER PUBLIC<br>◯ NGO HEALTH FACILITY<br>◯ OTHER NGO<br>◯ PRIVATE HOSPITAL<br>◯ PRIVATE CLINIC<br>◯ Private PHARMACY<br>◯ OTHER PRIVATE MEDICAL<br>◯ DRUG VENDORS/STORE<br>◯ Shop<br>◯ OTHER<br>◯ DO not KNOW<br>◯ NO RESPONSE
FFQ211c. Who tried to remove the implant?	◯ Self<br>◯ Friend/Relative<br>◯ Partner<br>◯ HEW<br>◯ Other professional healthcare provider, can't distinguish<br>◯ No one tried<br>◯ No response
FFQ212. Why were you not able to have your implant removed?<br>*Select all that apply*	◯ Facility not open<br>◯ Qualified provider not available<br>◯ Provider attempted but could not remove the implant<br>◯ Provider refused<br>◯ Cost of removal services<br>◯ Travel cost

	◯ Provider counseled against removal<br>◯ Told to return on another day<br>◯ Referred elsewhere<br>◯ Other<br>◯ Do not know<br>◯ No response
FFQ213. When you stopped using the implant, where did you go to have your implant removed?<br>*Scroll to bottom to see all choices.*	◯ GOVT HOSPITAL<br>◯ GOVT HEALTH CENTER<br>◯ GOVT. HEALTH POST/HEW<br>◯ Health development army<br>◯ Public Pharmacy<br>◯ OTHER PUBLIC<br>◯ NGO HEALTH FACILITY<br>◯ OTHER NGO<br>◯ PRIVATE HOSPITAL<br>◯ PRIVATE CLINIC<br>◯ Private PHARMACY<br>◯ OTHER PRIVATE MEDICAL<br>◯ DRUG VENDORS/STORE<br>◯ Shop<br>◯ OTHER<br>◯ DO not KNOW<br>◯ NO RESPONSE
FFQ214. Who removed the implant?	◯ Self<br>◯ Friend/Relative<br>◯ Partner<br>◯ HEW<br>◯ Other professional healthcare provider, can't distinguish<br>◯ No one tried<br>◯ No response
Now I would like to ask you some questions about why you stopped using your recent family planning methods. We still start with the one that you used most recently.<br>RE: Use visual aid to show different methods and dates. Start with the most recently used method and work backwards. FFQ215 is repeated for all previous methods listed in CS2	

ERROR: Too many groups added.<br>*Remove this group by pressing your finger down on the small circle below and selecting 'remove group' from the menu that appears.*	
######	◯
FFQ215. Did you stop using ${cs2_method_name} because of any of the following reasons?	☐ Became pregnant while using<br>☐ Infrequent sex/husband away

RECORD ALL REASONS MENTIONED. Cannot select "Do Not Know" or "No response" with other options.	☐ Wanted to become pregnant<br>☐ Side effects you experienced<br>☐ Side effects you were worried about but did not experience<br>☐ Husband did not approve<br>☐ Other person did not approve<br>☐ Wanted more effective method<br>☐ No method available<br>☐ Lack of access / too far<br>☐ Costs too much<br>☐ Inconvenient to use<br>☐ Fatalistic<br>☐ Difficult to get pregnant/menopausal<br>☐ Other<br>☐ Do not know<br>☐ No response
RE: For contraceptive use history, this method (number ${MSR_pos} entered) was marked as 'No response'. Therefore FFQ215 will be skipped for this method.<br>*Please proceed to the next screen.*	

CURRENT NON-USERS

FFQ217. Have you ever done anything or tried in any way to delay or avoid getting pregnant?	◯ Yes<br>◯ No<br>◯ No response
FFQ218. You said that you do not want any / anymore children and that you are not using a method to avoid pregnancy. Can you tell me the reason why you are not using a method to prevent pregnancy?<br>*PROBE: Any other reason? RECORD ALL REASONS MENTIONED. Cannot select "Do Not Know" or "No response" with other options. Cannot select "Not married" if 104 is "Yes, currently married". Scroll to the bottom to see all choices.*	☐ Not married<br>☐ Infrequent sex / not having sex<br>☐ Menopausal/Hysterectomy<br>☐ Subfecund / infecund<br>☐ Not menstruated since last birth<br>☐ Breastfeeding<br>☐ Husband/partner away<br>☐ Up to God / fatalistic<br>☐ Respondent opposed<br>☐ Husband / partner opposed<br>☐ Others opposed<br>☐ Religious reasons<br>☐ Does not know of a method<br>☐ Does not know where to get method<br>☐ Fear of side effects<br>☐ Health concerns<br>☐ Too far to get method<br>☐ Costs too much<br>☐ Preferred method not available<br>☐ No method available

	☐ Inconvenient to use<br>☐ Interferes with body's processes<br>☐ Other<br>☐ Do not know<br>☐ No response
FFQ219. Would you say that NOT using contraception is mainly your decision, mainly your husband/partner's decision or do you both decide together?	◯ Mainly respondent<br>◯ Mainly husband/partner<br>◯ Joint decision<br>◯ Other<br>◯ No response
FFQ220. Do you think you will use a contraceptive method to delay or avoid getting pregnant in the future?	◯ Yes<br>◯ No<br>◯ Do not know<br>◯ No response
FFQ221. When do you think you will start using a method?	◯ Months<br>◯ Years<br>◯ Soon / Now<br>◯ After the birth of this child<br>◯ Do not know<br>◯ No response
Enter in ${when_willuse_lab}<br>*Enter the age in ${when_willuse_lab}.*	
FFQ222. How old were you when you first used a method to delay or avoid getting pregnant? The respondent said she was ${age} years old at her last birthday.<br>*Enter the age in years. Enter -88 if respondent does not know. Enter -99 if there is no response.*	
FFQ223. How many living children did you have at that time, if any?<br>Note: the respondent said that she gave birth ${total_births} times in FFQ019.<br>*Enter -99 for no response*	
FFQ224. Have you used emergency contraception at any time in the last 12 months?<br>*PROBE: As an emergency measure after unprotected sexual intercourse women can take special pills at any time within three to five days to prevent pregnancy.*	◯ Yes<br>◯ No<br>◯ No response
FFQ225. In the last 12 months, were you visited by a health extension worker who talked to you about family planning?	◯ Yes<br>◯ No<br>◯ No response
FFQ226. In the last 12 months, have you attended a group family planning counseling session with a provider?	◯ Yes<br>◯ No<br>◯ No response

FFQ227. In the last 12 months, have you visited a health facility for care for yourself or your children?<br>*For any health services*	◯ Yes<br>◯ No<br>◯ No response
FFQ228. Did any staff member at the health facility speak to you about family planning methods?	◯ Yes<br>◯ No<br>◯ No response

FFQ229. In the last few months have you:	
A. Heard about family planning on the radio?	◯ Yes<br>◯ No<br>◯ No response
B. Seen anything about family planning on the television?	◯ Yes<br>◯ No<br>◯ No response
C. Read about family planning in a newspaper or magazine?	◯ Yes<br>◯ No<br>◯ No response
D. Received text message about family planning on a mobile phone	◯ Yes<br>◯ No<br>◯ No response
E. Seen anything on social media about family planning (Facebook, Viber, Twitter, WhatsApp etc)	◯ Yes<br>◯ No<br>◯ No response

FFQ230. Do you know if there is a law on abortion in Ethiopia?	◯ Yes<br>◯ No<br>◯ No response
FFQ231. Under which circumstances it is legal to have an abortion in Ethiopia?<br>*Read out the responses*	☐ In instances of rape<br>☐ When pregnancy is a risk to the life of the mother and/or fetus<br>☐ When fetus has been diagnosed with an incurable disease or serious deformity<br>☐ When pregnant woman is incapacitated/physically or mentally unfit to be a mother<br>☐ No circumstances<br>☐ Do not know<br>☐ No response
FFQ232. Do you know where a woman can access facility-based abortion services in the community where you live?	◯ Yes<br>◯ No<br>◯ No response

Section 5 – Partner

Now I would like to ask you some questions about your husband/partner.

FFQ250. Before you started using ${current_recent_method_lab}, had you discussed the decision to delay or avoid pregnancy with your husband/partner?	◯ Yes<br>◯ No<br>◯ Do not know<br>◯ No response
FFQ251. How does your husband/partner feel about family planning?	◯ He disapproves of it<br>◯ He does not care<br>◯ He is ok with it<br>◯ Do not know<br>◯ No response
FFQ252. Does/did your husband/partner know that you are/were using ${current_recent_method_lab}?	◯ Yes<br>◯ No<br>◯ Do not know<br>◯ No response
FFQ253. Did you talk with your partner about using your ${current_recent_method_lab} before you started using, after you started using, or you have not talked about it?	◯ Before<br>◯ After<br>◯ No discussion<br>◯ No response
FFQ254. Why have you not discussed your family planning use with your husband/partner?<br>*Select all that apply—do not read options aloud*	☐ It does not concern him<br>☐ There might be negative consequences<br>☐ He does not know about FP<br>☐ Other<br>☐ No response

FFQ255. In the past 12 months, has your husband/partner:	
A.Told you not to use any family planning	◯ Yes<br>◯ No<br>◯ No response
B. Said he would leave you if you didn't get pregnant	◯ Yes<br>◯ No<br>◯ No response
C. Told you he would have a baby with someone else if you didn't get pregnant	◯ Yes<br>◯ No<br>◯ No response
D. Took away your family planning or kept you from going to the clinic to get family planning	◯ Yes<br>◯ No<br>◯ No response

E. Hurt you physically because you did not agree to get pregnant	◯ Yes<br>◯ No<br>◯ No response

Section 6 - Empowerment/Norms

Now I'm going to ask you a series of statements about family planning. For each, please tell me how strongly you agree or disagree with the statement. Some will seem similar but we would like you to consider each one as different.

We can pause at any time. If you do not feel comfortable answering any of the statements, let me know and I will move onto the next statement.

FFQ256. If I use family planning, my husband/partner may seek another sexual partner.	◯ Strongly agree<br>◯ Somewhat agree<br>◯ Neither agree nor disagree<br>◯ Somewhat disagree<br>◯ Strongly disagree<br>◯ No response
FFQ257. If I use family planning, I may have trouble getting pregnant the next time I want to.	◯ Strongly agree<br>◯ Somewhat agree<br>◯ Neither agree nor disagree<br>◯ Somewhat disagree<br>◯ Strongly disagree<br>◯ No response
FFQ258. There could be/will be conflict in my relationship/marriage if I use family planning.	◯ Strongly agree<br>◯ Somewhat agree<br>◯ Neither agree nor disagree<br>◯ Somewhat disagree<br>◯ Strongly disagree<br>◯ No response
FFQ259. If I use family planning, my children may not be born normal.	◯ Strongly agree<br>◯ Somewhat agree<br>◯ Neither agree nor disagree<br>◯ Somewhat disagree<br>◯ Strongly disagree<br>◯ No response
FFQ260. If I use family planning, my body may experience side effects that will disrupt my relations with my husband/partner.	◯ Strongly agree<br>◯ Somewhat agree<br>◯ Neither agree nor disagree<br>◯ Somewhat disagree<br>◯ Strongly disagree<br>◯ No response
FFQ261. It is acceptable for a woman to use family planning before she has children	◯ Strongly agree<br>◯ Somewhat agree<br>◯ Neither agree nor disagree

	◯ Somewhat disagree<br>◯ Strongly disagree<br>◯ No response
FFQ262. Women who use family planning are considered promiscuous	◯ Strongly agree<br>◯ Somewhat agree<br>◯ Neither agree nor disagree<br>◯ Somewhat disagree<br>◯ Strongly disagree<br>◯ No response
FFQ263. Couples who use family planning are financially responsible	◯ Strongly agree<br>◯ Somewhat agree<br>◯ Neither agree nor disagree<br>◯ Somewhat disagree<br>◯ Strongly disagree<br>◯ No response
FFQ264. Women should be the ones to decide about family planning	◯ Strongly agree<br>◯ Somewhat agree<br>◯ Neither agree nor disagree<br>◯ Somewhat disagree<br>◯ Strongly disagree<br>◯ No response
Now, I'd like to ask a few questions about your community.	
FFQ270. Do most, some, few, or no people in your community encourage women to deliver at a facility?	◯ Most people<br>◯ Some people<br>◯ Few people<br>◯ No people<br>◯ Do not Know<br>◯ No response
FFQ271. Do most, some, few, or no people in your community think it is acceptable to deliver with a traditional birth attendant?	◯ Most people<br>◯ Some people<br>◯ Few people<br>◯ No people<br>◯ Do not Know<br>◯ No response
FFQ272. Do most, some, few, or no people in your community encourage going to antenatal care?	◯ Most people<br>◯ Some people<br>◯ Few people<br>◯ No people<br>◯ Do not Know<br>◯ No response
FFQ273. Do most, some, few, or no people in your community encourage women to seek postnatal care?	◯ Most people<br>◯ Some people<br>◯ Few people<br>◯ No people

	◯ Do not Know<br>◯ No response

Section 7. Sexual Activity

Now I'm going to ask you a few sensitive questions about sexual activity. You do not have to answer these questions if you do not want to. We can pause at any time. If you do not feel comfortable answering any of the questions, let me know and I will either move onto the next statement or skip this section entirely.

CHECK FOR THE PRESENCE OF OTHERS. BEFORE CONTINUING, MAKE EVERY EFFORT TO ENSURE PRIVACY.

FFQ277. You stated that you were not currently married or living with a man, but are you currently in a relationship?	◯ Yes<br>◯ No<br>◯ Do not know<br>◯ No response
FFQ278. How long have you been in a relationship with your current partner?	◯ Months<br>◯ Years<br>◯ No response
Enter in ${inreship_duration_lab}	
FFQ279a. How old were you when you first had sexual intercourse?<br>*The respondent said she was ${age} years old at her last birthday. She has had ${total_births} live births. Enter the age in years. Enter -77 if she never had sex. Enter -88 if respondent does not know. Enter -99 for no response.*	
FFQ279b. You have entered that the respondent was ${first_intercourse_age} years old when she first had sexual intercourse. Is this what she said?<br>*Go back and correct FFQ279a if it is not correct.*	◯ Yes<br>◯ No
FFQ280. Have you resumed sexual activity since the birth of your most recent child?	◯ Yes<br>◯ No<br>◯ No response
FFQ281. When was the last time you had sexual intercourse?<br>*If less than 12 months ago, answer must be recorded in months, weeks, or days. Enter 0 days for today. You will enter a number for X on the next screen. Enter -99 for no response*	◯ Days ago<br>◯ Weeks ago<br>◯ Months ago<br>◯ Years ago<br>◯ No response
Enter in ${last_intercourse_lab}	
We understand that this interview may have raised some difficult issues. How are you feeling after we've asked these questions? Would you like to speak with a trained care provider further? We can connect you to the local health center for follow-up care.	

END OF SURVEY Thank the respondent for her time<br>*The respondent is finished, but there are still 3 more questions for you to complete outside the home*	
U. Location<br>*Take a GPS point near the entrance to the household. Record location when the accuracy is smaller than 6m. GPS coordinates can only be collected when outside.*	
V. Did you have to step away from the respondent's home to take the GPS reading?	◯ Yes<br>◯ No
QUESTIONNAIRE RESULT	
W. How many times have you visited this household to interview this female respondent?	◯ 1st time<br>◯ 2nd time<br>◯ 3rd time
X. What language was this interview conducted in?	◯ English<br>◯ Amharic<br>◯ Afan Oromo<br>◯ Tigrigna<br>◯ Sidamigna<br>◯ Wolayitigna<br>◯ Afar<br>◯ Somali<br>◯ Kefigna<br>◯ Other
Y. Was a translator used for this interview?	◯ Yes<br>◯ No
Z. Questionnaire result<br>*Record the result of the Female Questionnaire*	◯ Completed<br>◯ Not at home<br>◯ Postponed<br>◯ Refused<br>◯ Partly completed<br>◯ Incapacitated<br>◯ Respondent death<br>◯ Respondent moved<br>◯ Household moved
